# Application of Deep Learning on Millimeter-Wave Radar Signals: A Review

**DOI:** 10.3390/s21061951

**Published:** 2021-03-10

**Authors:** Fahad Jibrin Abdu, Yixiong Zhang, Maozhong Fu, Yuhan Li, Zhenmiao Deng

**Affiliations:** Department of Information and Communication Engineering, School of Informatics, Xiamen University, Xiamen 361005, China; fajad03@gmail.com (F.J.A.); maozhong@gmail.com (M.F.); yuhanlily@gmail.com (Y.L.); dzm_ddb@xmu.edu.cn (Z.D.)

**Keywords:** automotive radars, object detection, object classification, deep learning, multi-sensor fusion, datasets, autonomous driving

## Abstract

The progress brought by the deep learning technology over the last decade has inspired many research domains, such as radar signal processing, speech and audio recognition, etc., to apply it to their respective problems. Most of the prominent deep learning models exploit data representations acquired with either Lidar or camera sensors, leaving automotive radars rarely used. This is despite the vital potential of radars in adverse weather conditions, as well as their ability to simultaneously measure an object’s range and radial velocity seamlessly. As radar signals have not been exploited very much so far, there is a lack of available benchmark data. However, recently, there has been a lot of interest in applying radar data as input to various deep learning algorithms, as more datasets are being provided. To this end, this paper presents a survey of various deep learning approaches processing radar signals to accomplish some significant tasks in an autonomous driving application, such as detection and classification. We have itemized the review based on different radar signal representations, as it is one of the critical aspects while using radar data with deep learning models. Furthermore, we give an extensive review of the recent deep learning-based multi-sensor fusion models exploiting radar signals and camera images for object detection tasks. We then provide a summary of the available datasets containing radar data. Finally, we discuss the gaps and important innovations in the reviewed papers and highlight some possible future research prospects.

## 1. Introduction

Over the last decade, autonomous driving and Advanced Driver Assistance Systems (ADAS) have been among the leading research domains explored in deep learning technology. Important progress has been realized, particularly in autonomous driving research, since its inception in 1980 and the DARPA urban competition in 2007 [1,2]. However, up to now, developing a reliable autonomous driving system remained a challenge [3]. Object detection and recognition are some of the challenging tasks involved in achieving accurate, robust, reliable, and real-time perceptions [4]. In this regard, perception systems are commonly equipped with multiple complementary sensors (e.g., camera, Lidar, and radar) for better precision and robustness in monitoring objects. In many instances, the complementary information from those sensors is fused to achieve the desired accuracy [5].

Due to the low cost of the camera sensors and the abundant features (semantics) obtained from them, they have now established themselves as the dominant sensors in 2D object detection [6,7,8,9,10] and 3D object detection [11,12,13,14] using deep learning frameworks. However, the camera’s performance is limited in challenging environments such as rain, fog, snow, dust, and strong/weak illumination conditions. While Lidar can provide depth information, they are relatively expensive.

On the other hand, radars can efficiently measure the range, relative radial velocity, and angle (i.e., both elevation and azimuth) of objects in the ego vehicle surroundings and are not affected by the change of environmental conditions [15,16,17]. However, radars can only detect objects in their environment within their measuring range, but they cannot provide the category of the object detected (i.e., a vehicle or pedestrian). Additionally, radar detections are relatively too sparse compared to Lidar point clouds [18]. Hence, it is an arduous task to recognize/classify objects using radar data. Based on the sensor’s comparative working conditions, as mentioned earlier, we can deduce that they complement one another, and they can be fused to improve the performance and robustness of object detection/classification [5].

Multi-sensor fusion refers to the technique of combining different pieces of information from multiple sensors to acquire better accuracy and performance that cannot be attained using either one of the sensors alone. Readers can refer to [19,20,21,22] for detailed discussions about multi-sensor fusion and related problems. Based on the conventional fusion algorithms using radar and vision data, a radar sensor is mostly used to make an initial prediction of objects in the surroundings with bounding boxes drawn around them for later use. Then, machine learning or deep learning algorithms are applied to the bounding boxes over the vision data to confirm and validate the presence of earlier radar detections [23,24,25,26,27,28]. Moreover, other fusion methods integrate both radar and vision detections using probabilistic tracking algorithms such as the Kalman filter [29] or particle filter [30], and then track the final fused results appropriately.

With the recent advances in deep learning technology, many research domains such as signal processing, natural language processing, healthcare, economics, agriculture, etc. are adopting it to solve their respective problems, achieving promising results [20]. In this respect, a lot of studies have been published over the recent years, pursuing multi-sensor fusion with various deep convolutional neural networks and obtaining a state-of-the-art performance in object detection and recognition [31,32,33,34,35]. The majority of these systems concentrate on multi-modal deep sensor fusion with cameras and Lidars as input to the neural network classifiers, neglecting automotive radars, primarily due to the relative availability of public accessible annotated datasets and benchmarks. This is despite the robust capabilities of radar sensors, particularly in adverse or complex weather situations where Lidars and cameras are largely affected. Ideally, one of the reasons why radar signals are rarely processed with deep learning algorithms has to do with their peculiar characteristics, making them difficult to be fed directly as input to many deep learning frameworks. Besides, the lack of open-access datasets and benchmarks containing radar signals have contributed to the fewer research outputs over the years [18]. As a result, many researchers self-developed their own radar signal datasets to test their proposed algorithms for object detection and classification using different radar data representations as inputs to the neural networks [36,37,38,39,40]. However, as these datasets are inaccessible, comparisons and evaluations are not possible.

Over the recent years, some radar signal datasets are being reported for public usage [41,42,43,44]. As a result, many researchers have begun to apply radar signals as inputs to various deep learning networks for object detection [45,46,47,48], object segmentation [49,50,51], object classification [52], and their combination with vision data for deep-learning-based multi-modal object detection [53,54,55,56,57,58]. This paper specifically reviewed the recent articles on deep learning-based radar data processing for object detection and classification. In addition, we reviewed the deep learning-based multi-modal fusion of radar and camera data for autonomous driving applications, together with available datasets being used in that respect.

We structured the rest of the paper as follows: Section 2 contains an overview of the conventional radar signal processing chain. An in-depth deep learning overview was presented in Section 3. Section 4 provides a review of different detection and classification algorithms exploiting radar signals on deep learning models. Section 5 reviewed the deep learning-based multi-sensor fusion algorithms using radar and camera data for object detection. Datasets containing radar signals and other sensing data such as the camera and Lidar are presented in Section 6. Finally, discussions, conclusions, and possible research directions are given in Section 7.

## 2. Overview of the Radar Signal Processing Chain

This section describes a brief overview of radar signal detection processes. Specifically, we discuss the range and the velocity estimation of different kinds of radar systems, such as Frequency Modulated Continuous Wave (FMCW), Frequency Shift Keying (FSK), and Multiple Frequency Shift Keying (MFSK) waveforms commonly employed in automotive radars.

### 2.1. Range, Velocity, and Angle Estimation

Radio Detection and Ranging (Radar) technology was first introduced around the 19th century, mainly targeting military and surveillance-related security applications. Interest in radar usage has now expanded over the last couple of years, particularly towards commercial, automotive, and industrial applications. The fundamental task of a radar system is to detect the targets in their surroundings and, at the same time, estimate their associated parameters, such as the range, radial velocity, azimuth angle, etc. The range and radial velocity measurements are largely dependent upon the time delay and Doppler frequency estimation accuracy, respectively.

This system usually emits an electromagnetic wave signal and then receives the reflections of those waves reflected by the targets along its propagation path [59]. Radar sensors generally transmit either continuous waveform or short sequences of pulses in the majority of radar applications. Therefore, according to radar system waveforms, radars are conventionally divided into two general categories: pulse and continuous wave (CW) radars with or without modulation.

Pulse radar transmits sequences of short pulse signals to estimate both the range and radial velocity of a moving target. The distance of the target from the radar sensor is calculated using the time delay that elapses between the transmitted and the intercepted pulse. In order to achieve better accuracy, shorter pulses are employed, while, to attain a better signal-to-noise ratio, longer pulses are necessary.

On the other hand, a CW radar operates by transmitting a constant unmodulated frequency to measure the target radial velocity but without range information. The transmitted signal from the CW radar antenna with a particular frequency is intercepted after it is reflected back from the target, with the change in its frequency known as the Doppler frequency shift. The velocity information is estimated based on the Doppler effect exhibited by the motion between the radar and the target. However, CW cannot measure the target range, which is one of its drawbacks.

The linear frequency modulated continuous (LFMCW) waveform is another important radar waveform scheme. Unlike CW, the transmitted waveform signal frequency is modulated to simultaneously estimate the target’s range and radial velocity with high resolution. Most of the modern-day automotive radars operate based on the FMCW modulation scheme, and it has been extensively studied in the literature [60,61]. They are gaining more popularity recently, as they are among the leading sensing components employed in applications like adaptive cruise control (ACC), autonomous driving, industrial applications, etc. Their main benefit is the ability to measure the range and radial velocity of moving objects simultaneously.

**Figure 1:**
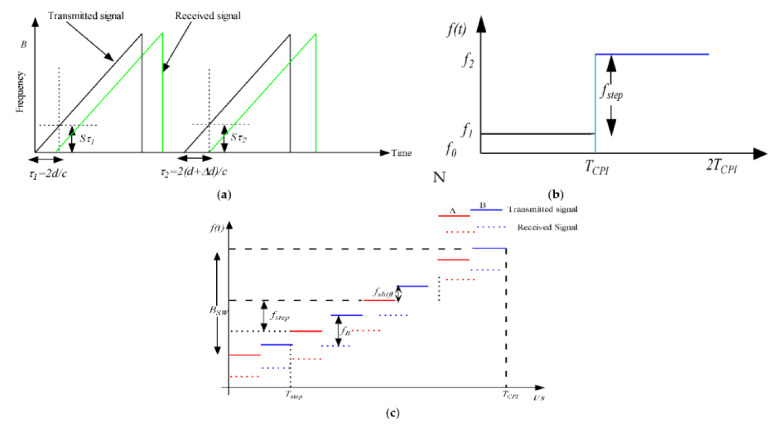
Range and velocity estimation schemes. (**a**) linear frequency modulated continuous waveform (LFMCW) scheme [52], (**b**) Frequency Shift Keying (FSK) waveform scheme [67], and (**c**) Multiple Frequency Shift Keying (MFSK) waveform system [68].

As depicted in Figure 1a, the FMCW transmits sequences of the linear frequency modulated signal (LFM), also called chirp signal, which increases linearly with time, within a bandwidth range of up to 4 GHz and a carrier frequency of 79 GHz [62], then receives the reflected signals that bounce back from the targets. The received signals are mixed with the transmitted signals (chirp) in a mixer at the receiving end to obtain another frequency called the beat frequency signal, given by:

(1) fb=2dsc

where d is the distance of the object from the radar, s is the slope of the chirp signal, and c is the speed of light.

Using this frequency, we can infer the distance of the target to the radar sensor. A fast Fourier transform (FFT) (range FFT) is usually performed on the beat frequency signal to convert it to the frequency domain, thereby separating the individual peaks of the resolved objects. The range resolution of this procedure partly depends on the bandwidth B of the FMCW system [60], given by:

(2) $$\partial_{res}=\frac{c}{2B}$$

The phase information of the beat signal is exploited to estimate the velocity of the target. As shown in Figure 1, the object motion Δd in relation to the radar results in a beat frequency shift, given by:

(3) $$\Delta f_{b}=\frac{2s\Delta d}{c}$$

over the received signal and a phase shift, given by [60]:

(4) $$\Delta \phi_{v}=2\pi f_{c}\frac{2\Delta d}{c}=\frac{4\pi vT_{c}}{\lambda }$$

where v is the object radial velocity, $f_{c}$ is the center frequency, $T_{c}$ is the chirp duration, and λ is the wavelength.

Since the phase shift of mm-wave signals is much more sensitive to the target object movements than the beat frequency shift, the velocity FFT is usually conducted across the chirps to generate the phase shift and then converted to the velocity afterward. The expression for the velocity resolution $\partial v_{res}$ can be represented as [61]:

(5) $$\partial v_{res}=\frac{\lambda }{2T_{f}}=\frac{\lambda }{2LT_{c}}$$

where L is the number of chirps in one frame, and $T_{f}$ is the frame period.

To estimate the target’s position in space, the target’s azimuth and elevation angles are calculated by processing the received signals using array processing techniques. The most typical procedures include the digital beamforming [63], phase comparison monopulse [61], and the Multiple Signal Classification (MUSIC) algorithm [64].

However, according to an FFT algorithm, the azimuth angle of a moving object is obtained by conducting a fast Fourier transform (Angle FFT) on the spatial dimension across the receiver antennas. The expression for the velocity resolution $\partial v_{res}$ can be represented as [61]:

(6) $$\partial \theta =\frac{\lambda }{N_{RX}h\cos\theta }$$

where $N_{RX}$ is the number of the receiver antennas, θ is the azimuth angle between the distant object to the radar position, and h is the distance between the receiver antenna pairs.

The conventional linear frequency modulation (LFMCW) waveform scheme explained earlier delivers the desired range and velocity resolution. However, it usually encounters ambiguities in multi-target situations during the range and velocity estimations, which is also referred to as the ghost target problem. One of the most straightforward approaches to address this problem is applying multiple chirp signals (i.e., multiple chirp continuous waves, each with a different frequency of modulation, are transmitted) [65]. However, this method will also lead to another issue, as it increases the measurement time.

In this regard, many other waveforms have been proposed by the research community to overcome one issue to another, such as Frequency Shift Keying (FSK) [66], Chirp Sequence [67], and, Multiple Frequency Shift Keying (MFSK) [68], to mention a few. However, it must be emphasized that selecting a specific radar waveform to be utilized in any form of radar system has always been a critical parameter dictating the performance. It usually depends on the role, purpose, or mission of the radar application.

For instance, as an alternative to the FMCW method, the FSK waveform can provide a significant range and velocity resolution while simultaneously withstanding ghost target ambiguities. Its only drawback is that it does not resolve the target in a range direction. This system transmits two discrete frequency signals (i.e., $F_{A}$ and $F_{B}$) sequentially in an intertwined passion within each $T_{CPI}$ time duration, as shown in Figure 1b. The difference between these two frequencies is called a step frequency and is defined as $f_{step}=F_{B}-F_{A}$. The step frequency is very small and is selected irrespective of the desired target range measured.

In this scheme, the receive echo signals are first down-converted into a baseband signal using the transmitted carrier frequency signal via a homodyne receiver and then sampled N times afterward. The output of the baseband signal conveys the Doppler frequency generated by the moving objects. A Fourier transform is conducted on the time-discrete receive signal for each coherent processing interval (CPI) within the $T_{CPI}$, and then, moving targets are detected after CFAR with an amplitude threshold. Suppose the frequency step ($f_{step}$) is maintained as minimal as possible regarding the intertwined transmitted signals $F_{A}$ and $F_{B}$. In that case, the Doppler frequencies obtained from the baseband signal outputs should be roughly the same, while the phase information changes at the spectrum’s peak. In this way, the moving object’s range is estimated using the phase difference’s peak ($\Delta \varphi =\varphi_{B}-\varphi_{A}$), as shown in Equation seven based on [66]:

(7) $$R=-\frac{c\Delta \varphi }{4\pi \cdot f_{step}}$$

where $\Delta \varphi =\varphi_{B}-\varphi_{A}$ is the phase difference measurement of the Doppler spectrum peak, R the range of the moving target, and c is the speed of light, while $f_{step}$ is a step frequency.

However, this scheme’s main drawback is that it cannot differentiate two targets with the same speed along the range dimension or when multiple targets are static. This is because multiple targets cannot be separated with phase information.

In the MFSK waveform scheme, the LFM and FSK waveforms combination is exploited to provide the range and radial velocity estimation of the target efficiently while, at the same time, avoiding the individual drawbacks of LFM and FSK [68]. The transmission signal waveform is a stepwise frequency modulated signal. In this case, the transmit waveform uses two linearly frequency modulated signals arranged in a sequence (e.g., ABABAB) with the same bandwidth and slope separated by a small frequency shift $f_{shift}$, as depicted in Figure 1c. Like in the case of FSK and LFM, the receive echo signals are down-converted to the baseband and sampled over each frequency step. Both signal sequences A and B are processed individually using the FFT and CFAR processing algorithms.

Due to the coherent measurement procedure in both sequences A and B, the phase information defined by $\Delta \varphi =\varphi_{B}-\varphi_{A}$ is used to estimate the target range and radial velocity. Analytically, the measured phase difference can be defined as given in [67] by Equation eight. Hence, it can be seen that MFSK cannot encounter the ghost target problem that is present in the LFM system.

(8) $$\Delta \varphi =\frac{\pi }{N-1}\cdot \frac{v}{\Delta v}.4\pi R\cdot \frac{f_{shift}}{c}$$

where N defines the number of frequency shifts for each sequence A and B, $f_{shift}$ is the frequency shift, c is the speed of light, and R is the range of the target.

Generally, selecting a radar waveform in a radar system design has always remained a challenging concern. It largely depends on many aspects, such as the role, purpose, and mission of the radar application. As such, a discussion about them is out of this study’s scope; however, more information can be found in [65,66,67,68,69].

### 2.2. Radar Signal Processing and Imaging

**Figure 2:**
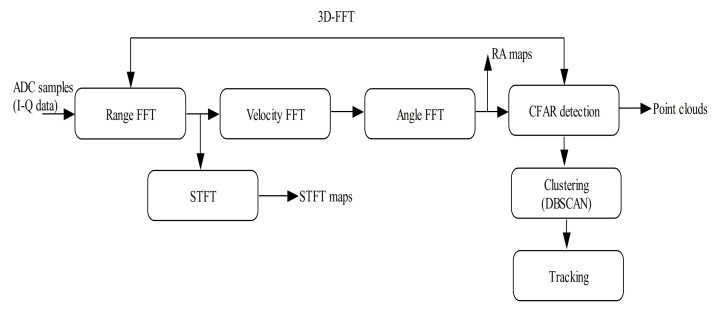
Radar signal processing and imaging. Adapted from [52].

The complete procedure is depicted in Figure 2, which consists of seven functional processing blocks. A fast Fourier transform is usually conducted over the 3D tensor to resolve the object range, velocity, and angle. In the beginning, the received radar signals (ADC samples) within a single coherent processing interval (CPI) are stored in matrix frames creating a 3D radar cube with three different dimensions, including fast time (chirp index), slow time (chirp sampling), and the phase dimension (TX/RX antenna pairs). Then, an unambiguous range–velocity estimation is the second processing stage, which is achieved via a 2D-FFT processing scheme on the 3D radar cube. Usually, the range FFT is first executed on an ADC time–domain signal to estimate the range. The second FFT (velocity FFT) is then performed across the chirps to estimate the relative radial velocity.

After these two FFT stages, a 2D map of velocity/range points is obtained, with higher amplitude values indicating the target candidate. Further processing is required to identify the real target against clutter. In order to create the Range-Velocity-Azimuth map, a third FFT scheme (Angle FFT) is executed over the maximum Doppler peaks of each range bin. The complete procedure represents a 3-dimensional FFT (including the range FFT, velocity FFT, and angle FFT). Similarly, a short-time Fourier transform (STFT) over the range FFT output can create the spectrogram, illustrating the object’s velocity.

The fourth stage is the target detection scheme, mainly performed using CFAR algorithms applied to the FFT outputs. A CFAR detection algorithm is applied to measure the noise within the target vicinity and then provide a more accurate target detection. The CFAR technique was proposed in 1968 by Fin and Johnson [70]. Instead of using a fixed threshold during target detection, they offered a variable threshold, which is adjusted by considering the noise variance in each cell’s neighborhood. However, at the moment, there are many different CFAR algorithms published with various ways of computing the threshold, such as the cell averaging (CA), smallest of selection (SO), greatest of selection (GO), and the ordered statistic (OS) CFAR [71,72]. Moreover, the 3D point clouds are generated by conducting an angle FFT on the CFAR detection obtained over the range–velocity bins.

A DBSCAN is also used to cluster the detected targets into groups in order to differentiate multiple targets [73]. Target tracking is the final stage in the radar signal processing chain, where algorithms such as the Kalman filter track the target position and target trajectory to obtain a smoother estimation.

## 3. Overview of Deep Learning

This section provides an overview of the current neural network frameworks widely employed in computer vision and machine learning-related fields that could also be applied for processing radar signals. This spans across different models on object detection and classification.

Over the last decade, computer vision and machine learning have seen tremendous progress using deep learning algorithms. This is driven by the massive availability of publicly accessible datasets, as well as the graphical processing units (GPUs) that enable the parallelization of neural network training [74]. Overwhelmed by its successes across different domains, deep learning is now being employed in many other fields, including signal processing [75], medical imaging [76], speech recognition [77,78], and much more challenging tasks in autonomous driving applications such as image classification and object detection [79,80].

However, before we dive into the deep learning discussion, it is important to talk about the traditional machine learning algorithm briefly, as it is the foundation of deep learning models. While deep learning and machine learning are specialized research fields in artificial intelligence, they have significant differences. Machine learning utilizes algorithms to analyze a given data, learn from it, and provide the possible decision based on what it has learned. One of the famous problems solved by machine learning algorithms is classification, where the algorithm provides a discrete prediction response. Usually, the machine algorithm uses feature extraction algorithms to extract notable features from the given input data and subsequently make a prediction using classifiers. Some examples of machine learning algorithms include symbolic methods such as support vector machines (SVM), Bayesian networks, decision trees, etc. and nonsymbolic methods such as genetic algorithms and neural networks.

On the other hand, a deep learning algorithm is structured based on the multiple layers of artificial neural networks, inspired according to the way neurons in the human brain function. Neural networks learn from the input data high-level feature representations, which are used to make intelligent decisions. Some common deep learning networks include deep convolutional neural networks (DCNNs), recurrent neural networks (RNNs), autoencoders, etc.

The most significant distinction between deep learning and machine learning is its performance, given the large amount of data available. However, when the training data is less, the deep learning performance is not that much. This is because they do need a large volume of datasets to learn perfectly. On the other hand, the classical machine learning methods perform significantly well with small data. Deep learning network functionality depends on powerful high-end machines. This is because deep learning models are composed of many parameters that require a longer time for training. Thus, they perform complex matrix multiplication operations that can be easily realized and optimized using GPUs, while, on the contrary, machine learning algorithms can work efficiently well even on low-end machines such as CPUs.

Another important aspect of machine learning is feature engineering, which utilizes domain knowledge to create feature extractors that minimize the complexity of the data and make the patterns in the data visible for the learning algorithm. However, this process is very challenging and time-consuming. Generally, the machine learning algorithm’s performance depends heavily on how precisely the features are identified and extracted. On the other hand, deep learning learns high-level features from its single end-to-end network. There is no need to put in any mechanisms to evaluate the features or understand what best represents the input data. In other words, deep learning does not need feature engineering, as the features are extracted automatically. Hence, deep learning eliminates the need for developing a new feature extraction algorithm for every problem.

### 3.1. Machine Learning

Machine learning is one of the new emerging disciplines that are now widely applied in the fields of science, engineering, medicine, etc. It is a subset of artificial intelligence that relies on computational statistics to produce a model showcasing the relations between the input and output data. Therefore, the system uses mathematical models to learn significant high-dimensional data structure (i.e., how to perform a particular task) from a given data and make decisions/predictions based on the learned information. The learning method can be divided into three categories—namely, supervised, unsupervised, and reinforcement learning. Classification and regression are the most typical tasks performed by machine learning algorithms. To solve a classification problem, the model is required (or task) to find which of the categories (k) an input corresponds to. Therefore, classification is required to discriminate an object from the list of all other object categories.

The first stage in the machine learning algorithm is feature extraction. The input data is processed and transformed into high-dimensional representations (i.e., features) that contain the most significant information from the objects, discarding irrelevant information. Shift, HOG, haar-like features, and Gaussian mixture models are some of the most widely traditional machine learning techniques employed for feature extraction. After the learning procedure, a decision can be achieved using classifiers. In most cases, Naïve Bayes, K-Nearest neighbor, and support vector machines (SVM) are the commonly exploited classifiers.

Machine learning is also used to learn important data structures from the radar data acquired for different moving targets. Many papers have been presented in the literature for radar target recognition using machine learning methods [81,82,83]. For instance, the author of [81] presented a classification of airborne targets based on a supervised machine learning algorithm (SVM and Naïve Bayes). Airborne radar was used to provide the measurements of the aerial, sea surface, and ground moving targets. C. Abeynayake et al. [82] developed an automatic target recognition approach based on a machine learning algorithm applied to ground penetration radar data. Their system helps detect complex features that are relevant to a multitude of thread objects. In [83], a machine learning-based method for target detection using radar processors was proposed, where they compared the performance of the machine learning-based classifiers (random decision forest) with one of the deep learning algorithms (RNNs). The results of their approach demonstrated that machine learning classifiers could discriminate targets from clutter with good precision. The main disadvantage of the machine learning approach is that it requires the prior feature extraction procedure before the final decision-making. With the recent revolution brought about by deep learning technology due to the availability of huge data and bigger processing tools (i.e., GPUs), machine learning models are now being regarded as inferior in performance, though their computational complexity is lighter, and a high performance can be achieved with a small amount of training data.

### 3.2. Deep Learning

Deep learning belongs to the subsets of machine learning algorithms that can be viewed as an extension of artificial neural networks (ANNs) applied to row sensory data to capture and extract high-level features that can be mapped to the target variable. For example, given an image as the sensory data, the deep learning algorithm will extract the object’s features, like edges or texture, from the raw image pixels.

**Figure 3:**
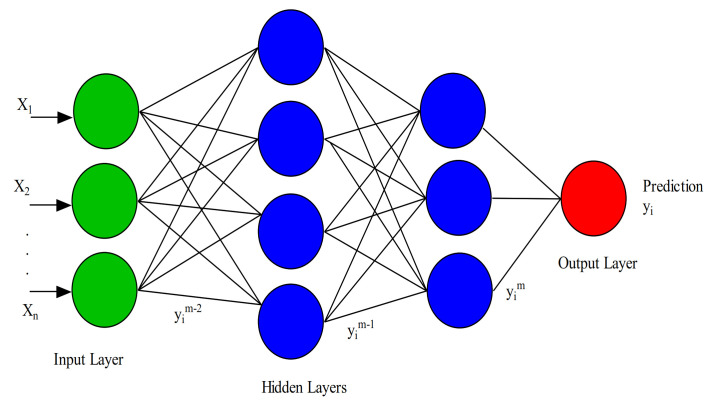
A simple structure of neural networks—input layer in green, output in red, and the hidden layers in blue.

In general, a deep learning algorithm consists of ANNs with at least one or more intermediate layers. The network is considered “deep” after stacking several intermediate layers of ANNs. The ANNs are responsible for transforming the low-level data to a higher abstracted level of representation. In this network, the first layer’s output is passed as input to the intermediate layers before producing the final output. Through this process, the intermediate layers enable the network to learn a nonlinear relationship between the inputs and outputs by extracting more complex features, as depicted in Figure 3. Deep learning models consist of many different components stacked together to form the main network model (e.g., convolution layers, pooling layers, fully connected layers, gates, memory cells, encoders, decoders, etc.), depending on the type of the network architecture employed (e.g., CNNs, RNNs, or autoencoders).

### 3.3. Training Deep Learning Models

Deep learning employs the Backpropagation algorithm to update the weights in each of the layers during the course of the learning process. The weights of the network are usually initialized randomly using small values. Given a training sample, the predictions are obtained based on the current weight’s values, and the outputs are compared with the target variable. An objective function is utilized to make the comparisons and estimate the error. The error obtained is fed back into the network for updating the network weights accordingly. More information on Backpropagation can be found in [84].

### 3.4. Deep Neural Network Models

Here, we provide an overview of some of the popular deep neural networks utilized by the research communities, which include the deep convolutional neural networks (DCNNs), recurrent neural networks (RNNs), long short-term memory (LSTM), encoder-decoder, and the generative adversarial networks (GANs).

#### 3.4.1. Deep Convolutional Neural Networks

Deep convolutional neural networks (DCNNs) are one of the most prominent deep learning models utilized by research communities, especially in the computer vision and related fields. DCNNs were first introduced by K. Fukushima [85], using the concept of a hierarchical representation of receptive fields from the visual cortex, as presented by Hubel and Wiesel. Afterward, Weibel et al. [86] proposed convolutional neural networks (CNNs) that share weights with temporal receptive fields and Backpropagation training methods. Later, Y. LeCun [87] presented the first CNN architecture for document recognition. The DCNN models typically accept 2D images or sequential data as the input.

**Figure 4:**
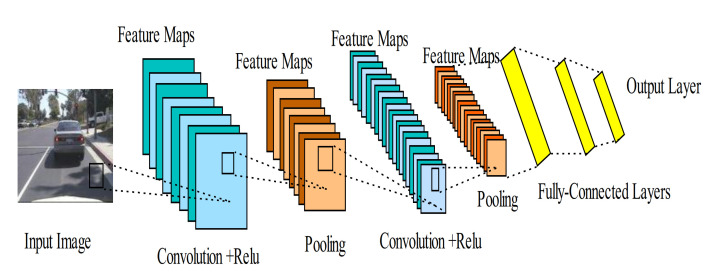
A simple deep convolutional neural network (DCNN) architecture. Adapted from [87].

A DCNN consists of several convolutional layers, pooling layers, nonlinear layers, and multiple fully connected layers that are periodically stacked together to form the complete network, as shown in Figure 4. Within the convolutional layers, CNN uses a set of filters (kernels) to convolve the input data (usually, a 2D image) and extract various feature maps. The nonlinear layers are mainly applied as activation functions to the feature maps. In contrast, the pooling operation is used for down-sampling the extracted features and learning invariant to small input translations. Max-pooling is the most commonly employed pooling method. Lastly, the fully connected layers are used to transform the feature maps into a feature vector. Stacking these layers together will form a deep multi-level layer network, with the higher layers being the composite of the lower layers. The network’s name was derived from the convolution operation that spread across all the layers in the whole network. A standard convolution operation in a simple CNN model involves the multiplication of 2D image I with a kernel filter K, as given in [87] and shown below:

(9) $$c\left(i,j\right)=\left(I*K\right)\left(i,j\right)=\sum_{m}\sum_{n}I\left(m,n\right)K\left(i-m,j-n\right)$$

For a better understanding, the process involved in the whole DCNN can be better represented mathematically if we express X as the input data of size m×n×d, with m×n representing the spatial level of X, and d as the number of channels. Similarly, if we assume a $j^{th}$ filter with its associated weights $w_{j}$ and bias $b_{j}$. Then, we can obtain the $j^{th}$ output associated with the convolutional layer, as given in [87]:

(10) $$y_{j}=\sum_{i=1}^{d}f(x_{i}*w_{j}+b_{j}),\;j=1,2,\ldots ,k$$

where the activation function $\left(.\right)$ is employed to improve the network nonlinearity; at the moment, ReLu [79] is the most commonly used activation function in the literature.

DCNNs have been the most employed deep learning-based algorithms over the last decade in many applications. This is due to their strong capability to explore the local connectivity from the input data based on its multiple combinations of convolution and pooling layers that automatically extract the features. Among the most popular DCNN architectures are Alex-Net [79], VGG-Net [88], Res-Net [89], Google-Net [90], Mobile-Net [91], and Dense-Net [92], to mention a few. Their promising performances achieved in image classification have led to their application in learning and recognizing radar signals. Furthermore, weight sharing and invariance to translation, scaling, rotation, and other transformations of the input data are essential in recognizing radar signals. Over the past few years, DCNNs have been employed to process various types of millimeter-wave radar data for object detection, recognition, human activity classification, and many more tasks [37,38,39,45,47], with excellent performance accuracy and efficiency. This is due to their ability to extract high-level abstracted features by exploiting the radar signal’s structural locality. Similarly, using DCNNs with the radar signal will allow us to extract features according to their frequency and pace. However, DCNNs cannot model sequential data from the human motion with temporal information, because every type of human activity consists of a specific spectral kind of posture.

#### 3.4.2. Recurrent Neural Networks (RNNs) and Long Short-term Memory (LSTM)

Recurrent neural networks (RNNs) are the type of network models designed specifically for processing sequential data. The output of RNNs constitutes the present inputs and the earlier outputs embedded in their hidden state h [93]. This is because they have a memory to store the earlier outputs that the multilayer perceptron neural networks lack. Equation (11) shows the update in the memory state according to [88]:

(11) $$h_{t}=f[Wx_{t}+Uh_{t-1}]$$

where f is a nonlinear transformation function such as tanh or ReLu, $x_{t}$ is the input to the network at a time t, $h_{t}$ represents the hidden state at a time t and can act as the memory of the network, and $h_{t-1}$ represents the previous hidden state. Similarly, U is a weight matrix that represents the RNN input-to-hidden connections, while W is the weight matrix representing the hidden-to-hidden recurrent connections.

**Figure 5:**
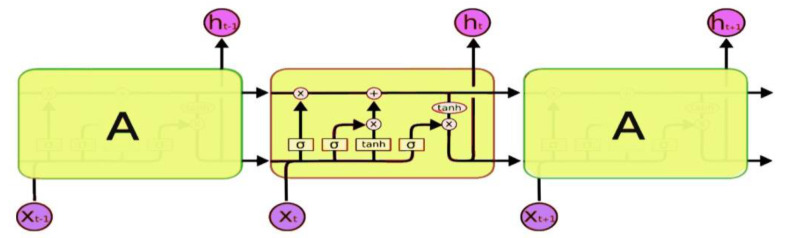
Long short-term memory (LSTM) architecture. Image source [96].

RNNs are also trained using the Backpropagation algorithm and can be applied in many areas, such as natural language processing, speech recognition, and time series prediction. However, RNNs suffered a deficiency called gradient instability, which means that, as the input sequence grows, the gradient vanishes or explodes. A long short-term memory (LSTM) network model was later proposed in [94] to overcome this problem and was then upgraded in [95]. LSTM adds a memory cell to the RNN networks to store each neuron’s state, thus preventing the gradient from exploding. LSTM architecture is shown in Figure 5, consisting of three different gates that control the memory cell’s data flow.

Unlike DCNNs, which only process input data of predetermined sizes, RNNs and the LSTM predictions increase with more available data. Their output prediction changes with time. Accordingly, they are sensitive to the change in the input data. For radar signal processing, especially human activity recognition, RNNs can exploit the radar signal temporal and spatial correlation characteristics, which is vital in human activity recognition [38].

As shown in Figure 5, LSTM has a chain-like structure, with repeated neural network blocks (yellow rectangles) having different structures. The neural network blocks are also called memory blocks or cells. Each cell A accepts input $x_{t}$ at a time t and output $h_{t}$, called the hidden state. Then two states are transferred to the next cell—namely, the cell state and the hidden state. These cells are responsible for remembering what is performed inside them while their manipulations are performed using gates (i.e., input gate, forget gate, and output gate) [95]. The input gate adds the information to the cell state. A forget gate is used to remove information from the cell state, while the output gate is responsible for creating a vector by applying a function (tanh) to the cell state and uses filters to regulate them before sending the output.

#### 3.4.3. Encoder-Decoder

The encoder-decoder network is the kind of model that uses a two-stage network to transform the input data into the output data. The encoder is represented by a function $z=g\left(x\right)$ that encodes the input information and transforms them into higher-level representations. Simultaneously, the decoder given by y=f(z) tries to reconstruct the output from the encoded data [97].

**Figure 6:**
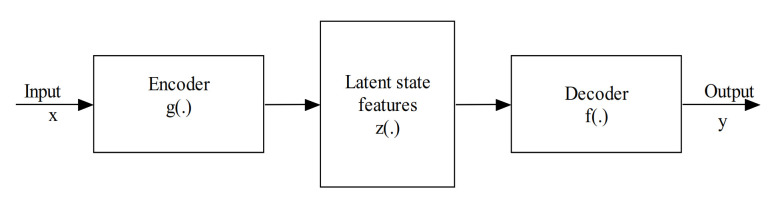
A simple encoder-decoder architecture.

The high-level representations used here literally refer to the feature maps that capture the essential discriminant information from the input data needed to predict the output. This model is particularly prevalent in image-to-image translation and natural language processing. Besides, this model’s training minimizes the error between the real and the reconstructed data. Figure 6 illustrates a simplified block diagram of the encoder-decoder model.

Some of the encoder-decoder model variants are stacked autoencoder (SAE), the convolutional autoencoder (CAE), etc. Many such models have been applied in many application domains, including radar signal processing [98]. For instance, because of the benefit of localized feature extraction, as well as the unsupervised pretraining technique, CAE has superior performance over DCNN for moving object classification. However, most of these models are based on fully connected networks, and they could not necessarily extract the structured features embedded in the radar data, especially those contained in the range cells of a high range resolution profile wideband radar (HRRP). This is because HRRP returned target scatterer distributions based on the range dimension.

#### 3.4.4. Generative Adversarial Networks (GANs)

**Figure 7:**
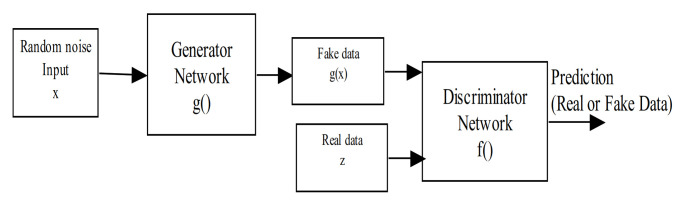
Generative adversarial network.

Generative Adversarial Networks (GANs) are among the most prominent deep neural networks in the generative models family [99]. They are made of two network blocks, a generator, and a discriminator, as shown in Figure 7. The generator usually receives a random noise as its input and processes it to produce the output samples that look similar to the data distribution (e.g., fake images). In contrast, the discriminator tries to compare the difference between the real data samples and those produced by the generator.

One of the major bottlenecks in applying deep learning models using radar signals is the lack of accessible radar datasets with annotations. Although labeling is one of the most challenging tasks in computer vision and its related applications, with the unsupervised generative models such as GANs, one could generate a huge amount of radar signal data and train it in an unsupervised manner, neglecting the need for laborious labeling tasks. In this regard, GANs have been used over the years for many applications, but very few studies have been performed using GANs and radar signal data [100]. However, GANs have a crucial issue regarding their training aspect, which sometimes leads to its collapse (instability). However, many of its variants have been proposed over the years to tackle this specific problem.

#### 3.4.5. Restricted Boltzmann Machine (RBM) and Deep Belief Networks (DBNs)

**Figure 8:**
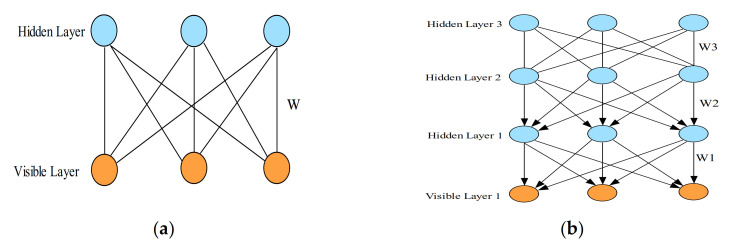
(**a**) Schematic of the Restricted Boltzmann Machine (RBM) architecture. (**b**) Schematic architecture of deep belief networks with one visible and three hidden layers [101].

Restricted Boltzmann Machine (RBM) has received increasing consideration over the recent years, as they have been adopted as the essential building components of deep belief networks (DBN) [101]. They are a specialized Boltzmann Machine (BM) with an added restriction of discarding all the connections within each visible or hidden layer, as shown in Figure 8a. Thus, the model is described as a bipartite graph. Therefore, an RBM can be described as a probabilistic model consisting of a visible layer (units) and a hidden layer (units) that extract a given data’s joint probability [102]. The two layers are connected using symmetrically undirected weights, while there are no intra-connections within either of the layers. The visible layer describes the input data (observable data) whose probability distribution is expected to be determined, while, on the other hand, the hidden layers are trained and expected to learn higher-order representations from the visible units.

The joint energy function of an RBM network according to the hidden and visible layers E(v,h) is determined using its weight and bias, as expressed in [102]:

(12) $$E(v,h;\theta )=-\sum_{i=1}^{V}\sum_{j=1}^{H}v_{i}h_{i}w_{ij}-\sum_{i=1}^{V}b_{i}v_{i}-\sum_{j=1}^{H}a_{j}h_{i}$$

where w represents the symmetric weight, and b and a denotes the bias of the visible unit $v_{i}$ and the hidden unit $h_{j}$, respectively. Therefore, $\theta =\left\{W,b,a\right\}$ and $v_{i},h_{j}\in \left\{0,1\right\}$.

The model allocates a joint probability distribution to each vector combination in the layers based on the energy function defined in [102] and given by:

(13) $$p\left(v,h\right)=\frac{1}{Z}\ell^{-E\left(v,h\right)}$$

where z is the normalization factor determined by adding up all the possible combinations of visible and hidden vectors, defined as:

(14) $$Z={\sum_{v,h}\ell }^{-E\left(v,h\right)}$$

Deep belief networks (DBN) are generative graphical deep learning models developed by R. Salakhutdinov and G.Hinton [103], in which they demonstrated that multiple RBMs could be stacked and trained in a specialized way (called the greedy approach). Figure 8b illustrates an example of three-layer deep belief networks. Unlike in the RBM model, a DBN only uses bidirectional connections (i.e., the same as in RBM) on its first top layer. In contrast, the subsequent layers use only top-down connections (bottom layers). The main reason behind this model’s recent interest is related to its new training principle called the layer-wise pretraining (i.e., the greedy method). Thus, DBN networks have recently been applied in many research domains, such as speech recognition, image classification, and audio classification.

The simple, most familiar application of the DBN model is feature extraction. The complexity of the DBN’s learning procedure is higher, as it learns the joint probability distribution of the output data. There is also a serious overfitting concern about DBN to the vanishing gradient, which changes the training from lower to higher network depth levels.

### 3.5. Object Detection Models

Object detection can be viewed as an act of identifying and localizing one or multiple objects from the given scene. This usually involves estimating the classification probability (labels) in conjunction with calculating the object’s location or bounding boxes. DCNN-based object detectors are grouped into two: the two-stage object detectors and the one-stage object detectors.

#### 3.5.1. One-Stage Object Detectors

This method uses only one single-stage network model to extract the feature maps used to obtain the classification scores and bounding boxes. Many unified one-stage models have been proposed in the literature. For instance, the earlier models include Single-Shot Multi-Box Detector (SSD) [7], which uses small CNN filters to predict multi-scale bounding boxes. This model is aimed at handling an object with different sizes. Yolo Object Detector [8] is the fastest among the single-stage family. It regresses the bounding boxes in conjunction with the classification score directly via a single CNN model.

#### 3.5.2. Two-Stage Object Detectors

Firstly, the object candidate region, also called Region of Interest (ROI) or Region Proposal (RP), is predicted from a given scene. The ROIs are then processed to acquire the classification score and the bounding boxes of the target objects. Examples of these types of object detectors are R-CNN [104], Fast-RCNN [105], Faster-RCNN [6], and Mask-R-CNN [9]. The region proposal generation ideally helped these types of models to provide better accuracy than one-stage detectors.

However, this comes with the disadvantage of huge, sophisticated training and high-inference time accrue, making them relatively slower than the one-stage counterpart. In contrast, one-stage object detectors are easier to train and faster for real-time applications.

## 4. Detection and Classification of Radar Signals Using Deep Learning Algorithms

This section provides an in-depth review of the recent deep learning algorithms that employ various radar signal representations for object detection and classification in both ADAS and autonomous driving systems. One of the most challenging tasks in using radar signals with deep learning models is representing the radar signals to fit in as inputs to the various deep learning algorithms.

**Figure 9:**
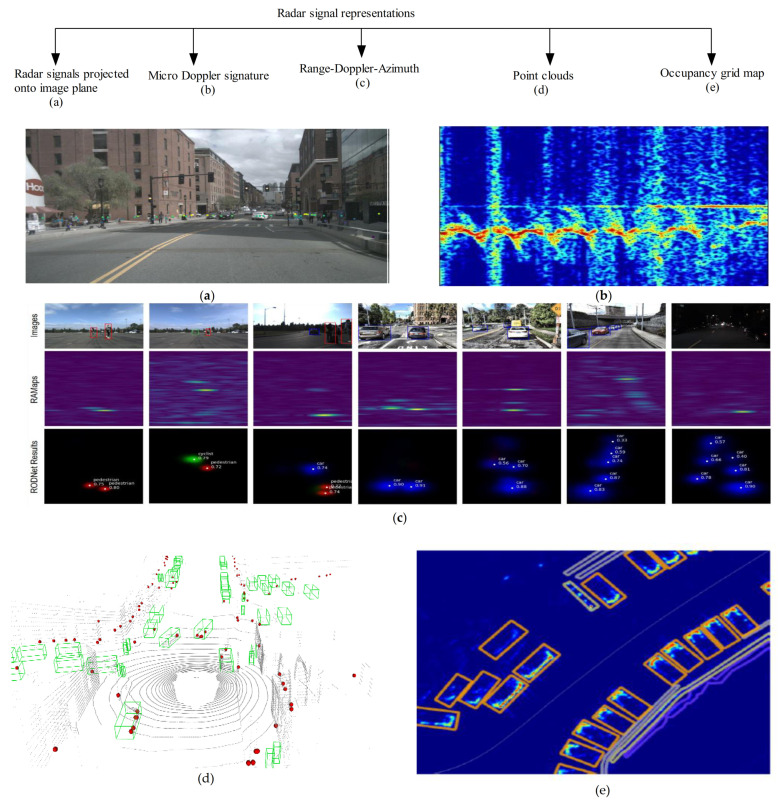
Example of different radar signal format representations. (**a**) Radar point clouds projected onto an image camera plane [58], with different colors depicting the depth information, (**b**) Spectrograms of a person walking away from the FMCW radar. (**c**) Samples of the Range-Doppler-Azimuth (RAMAPs) representations from the CRUW dataset [98], with the first row showing the images from the scene and the second row depicting their equivalent RAMAP tensors (d) Sample of Radar point clouds (red) with 3D annotations (green) and Lidar point clouds (grey) from the Nuscenes dataset. Image from [118]. And, (**e**) Example of a radar occupancy grid from a scene with multiple parked automobiles [119]. The white line represents the test vehicle driving path, and the rectangles represent the manually segmented objects along the grid.

In this respect, many radar data representations have been proposed over the years. These include radar occupancy grid maps, Range-Doppler-Azimuth tensor, radar point clouds, micro-Doppler signature, etc. Each one of these radar data representations has its pros and cons. With the recent availability of accessible radar data, many studies have begun to explore radar data to understand them extensively. Thus, we based our review article on this direction. Figure 9 illustrates an example of the various types of radar signal representations.

### 4.1. Radar Occupancy Grid Maps

For a host vehicle equipped with radar sensors and drives along a given road, radar sensors can collect data about its motion in that environment. At every point in time, radars can resolve the object’s radial distance, the azimuth angle, and the radial velocity that falls within its field of view. Distance and angle (both elevation and azimuth) entail more about the target’s relative position (orientation) concerning the ego vehicle coordinate system. Simultaneously, the target’s radial velocity obtained from the Doppler frequency shift will aid in detecting the moving targets.

Hence, based on the vehicle pose, radar return signals can be accumulated in the form of occupancy grid maps from which algorithms in machine learning and deep learning can be utilized to detect the objects surrounding the ego vehicle. In this way, both static and dynamic obstacles in front of the radar can be segmented, identified, and classified. The authors of [106] discussed different radar occupancy grid map representations. The grid map algorithm’s sole purpose is to determine the probability of whether each of the cells in the grids is empty or occupied.

Elfes reported the first occupancy grid map-based algorithm for robot perception and navigation [107]. In the beginning, most of the algorithms, especially in robotics, used laser sensor data. However, with the recent success of radar sensors, many occupancy grids employ radar data for different applications. In this case, assuming we have an occupancy grid map, $M_{t}=\left\{m_{1},m_{2}\ldots ,m_{n}\right\}$, consisting of N grid cells of $m_{i}$ that represent an environment with a 2D grid of equally spaced cells. Each of these cells is a random variable with a probability value of either [0, 1] expressing their occupancy states over time. For instance, if $M_{t}$ is a grid map representation for a time instance t, the cells are assumed to be mutually independent of one another. Then, the occupancy map can be estimated based on the posterior probability [107]:

(15) $$P\left(M_{t}\;|\;Z_{1:t},X_{1:t}\right)=\prod_{i}P\left(m_{i}\;|\;Z_{1:t},X_{1:t}\right)$$

where $P\left(m_{i}\;|\;Z_{1:t},X_{1:t}\right)$ is the inverse sensor model, and it represents the occupancy probability of the $i^{th}$ cell, $Z_{1:t}$ denotes the sensor measurement, and $X_{1:t}$ is the dynamic object pose from the ego vehicle.

A Bayes filter is typically used to calculate the occupancy value for each cell. Mainly, a posterior log formulation is used to integrate each of the new measurements for convenience.

Even though CNNs function extraordinarily well on images, they can also be tried and applied to other sensors that can yield image-like data [108]. The two-dimensional radar grid representations accumulated according to different occupancy grid map algorithms have already been exploited in deep learning domains for various autonomous system tasks, such as static object classification [109,110,111,112,113,114] and dynamic object classification [115,116,117]. In this case, the objects denote any road user within an autonomous system environment, like the pedestrian, vehicles, motorcyclists, etc.

For example, [109] is one of the earliest articles that employed machine learning techniques with a radar grid map. They proposed a real-time algorithm for detecting parallel and cross-parked vehicles using radar grid maps generated based on the occupancy grid reported by Elfes [107]. The candidate’s region was extracted, and two random forest classifiers were trained to confirm the parked vehicle’s presence. Subsequently, Lambacher et al. [110,111] presented a classification technique for static object recognition based on radar signals and DCNNs. The occupancy grid algorithm was used to accumulate the radar data into grid representations. All the occupancy grid cells were represented by a probability denoting whether it was occupied or not. Bounding boxes were labeled around each of the detected objects and applied to the classifiers as inputs. Bufler and Narayanan [112] also classified indoor targets with the aid of SVM. They generated their feature vectors using radar cross-entropy and observation angles from the simulated and measured objects.

The authors of [113] illustrate how to perform static object classification based on radar grid representation. Their work proves that semantic knowledge can be learned from the generated radar occupancy grids and accomplish cell-wise classification using CNN. L. Sless et al. [114] proposed an occupancy grid mapping using clustered radar data (point cloud). Ideally, the authors formulated their approach as a computer vision task in order to learn three semantic segmentation problems—namely, occupied, free, and unobserved spaces in front of or around vehicles. The main fundamental idea behind their proposed approach is the adoption of a deep learning model (i.e., encoder-decoder) to learn the occupancy grid mapping from the clustered radar data. They showed that their approach outperformed the classical filtering methods commonly used in the literature.

Usually, in the ideal case, the occupancy grid algorithm detects moving objects by removing moving objects based on their Doppler velocity. However, for complex systems, such as autonomous vehicle systems, both static and dynamic moving objects need to be detected simultaneously for the whole system’s efficacy. Hence, a radar grid map representation may not be suitable for dynamic objects, as other features have to be exploited in order to recognize dynamically moving objects like pedestrians. For dynamic road users such as vehicles, pedestrians, cyclists, etc., a grid map algorithm would require a longer time to be realized. This will not be good for applications like an autonomous system where latency is necessary.

Some authors, like [115,116], applied feature-based methods for classification. Schumann et al. [117] utilized a random forest classifier and long short-term memory (LSTM) based on radar data to classify dynamically moving objects. Feature vectors are generated from clustered radar reflections and fed to the classifiers. They found LSTM useful in their approach, as they were dealing with dynamic moving objects, since it is challenging to transform their radar signal into the image-like data needed by the CNN algorithms, while, for LSTM, successive feature vectors are grouped into a sequence.

The main problem with the radar grid map representations with regards to the deep learning and autonomous driving systems are:

- After the radar grid map generation, some significant information from the raw radar data may be lost, and thus, they cannot contribute to the classification task.
- The technique may result in a huge map, with many pixels in the grid map being empty, therefore adding more burden to the system complexity.

### 4.2. Radar Range-Velocity-Azimuth Maps

Having talked about radar grid representations in the previous section, as well as their drawbacks, especially in detecting moving targets. It will be essential to explore other ways to represent the radar data so that more information can be added to achieve a better performance. A radar image created via multidimensional FFT can preserve more informative data in the radar signal, as well as conforms to the required 2D grid data representation applicable to the deep learning algorithms like CNNs.

Many kinds of radar image tensors can be generated from the raw radar signals (ADC samples). This includes the range map, the Range-Doppler map, and the Range-Doppler-Azimuth map. A range map is a two-dimensional map that reveals the range profile of the target signal over time. Therefore, it demonstrates how the target range changes over time and can be generated by performing one-dimensional FFT on the raw radar ADC samples.

In contrast, the Range-Doppler map is generated by conducting 2D FFT on the radar frames. The first FFT (also called range FFT) is performed across samples in the time domain signal, while the second FFT (the velocity FFT) is performed across the chirps. In this way, a 2D image of radar targets is created that resolves targets in both range and velocity dimensions.

The Range-Doppler-Azimuth map is interpreted as a 3D data cube. The first two dimensions denote range–velocity, and the third dimension contains information about the target position (i.e., azimuth angle). The tensor is created by conducting 3-dimensional FFT (3D FFT), also known as the range FFT, the velocity FFT, and the angle FFT, on the radar return samples sequentially to create the complete map. Range FFT is performed on the time domain signal to estimate the range to the radar. Subsequently, velocity FFT is executed across the chirp’s frames to generate the Range-Doppler spectrum and then passed on to the CFAR detection algorithm to create a 2D sparse point cloud that can distinguish between real targets and the clutter. Finally, angle FFT is performed on the maximum Doppler peak of each range bin (i.e., detector Doppler), resulting in a 3D Range-Velocity-Azimuth map.

Most of the earlier studies using the Range-Doppler spectrums extracted from the automotive radar sensors performed either road user detection or classification using machine learning algorithms [36,120,121]. Reference [120] achieved pedestrian classification using a 24-GHz automotive radar sensor for city traffic application. Their system employed support vector machines, one of the most prominent machine learning algorithms, to discriminate pedestrians, vehicles, and other moving objects. Similarly, [121] presented an approach to detect and classify pedestrians and vehicles using a 24-GHz radar sensor with an intertwined Multi-Frequency Shift Keying (MFSK) waveform. Their system considered target features like the range profile and Doppler spectrum for the target recognition task.

S. Heuel and H. Rohling [36] presented a two-stage pedestrian classification system based on a 24-GHz radar sensor. In the first stage, they extracted both the Doppler spectrum and the range profile from the radar echo signal and fed it to the classifier. In the second stage, additional features were obtained from the tracking system and sent back to the recognition system to further improve the final system performance.

However, the techniques mentioned above based on machine learning require long time accumulations and feature selection to achieve a better performance from the handcrafted features learned on Range-Doppler maps. Due to the success achieved by deep learning algorithms in different tasks, such as image detection and classification, many researchers have now begun to apply it in their domains to benefit from its better performance. With enough training samples and GPU processors, deep learning provides a much better understanding than its machine learning counterparts.

The Range-Doppler-Azimuth spectrums extracted from automotive radar sensors have been used frequently as 2D image inputs to various deep learning algorithms for different tasks, ranging from obstacle detection to segmentation, classification, and identification in autonomous driving systems [122,123,124,125,126]. The authors of [122] presented a method to recognize objects in Cartesian coordinates using a high-resolution 300-GHz scanning radar based on deep neural networks. They applied a fast Fourier transform (FFT) on each of the received signals to obtain the radar image. Later, the radar image was converted from a polar radar coordinate to a Cartesian coordinate and used as an input into the deep convolutional neural network. Patel et al. [123] proposed an object classification based on a deep learning approach directly applied to automotive radar spectra for scene understanding. Firstly, they used a multidimensional FFT on the radar spectra to obtain the 3D Range-Velocity-Azimuth maps. Secondly, a Region of Interest (ROI) is extracted from the Range-Azimuth maps and used as an input to the DCNNs. Their approach could be seen as a potential substitute for conventional radar signal processing and has achieved better accuracy than the machine learning methods. This approach is particularly interesting, as most of the literature uses the full radar spectrum after the multidimensional FFT.

Similarly, Benco et al. [124] used radar signals to illustrate a deep learning-based vehicle detection system for autonomous driving applications. They represent the radar information as a 3D tensor using the first two spatial coordinates (i.e., Range-Azimuth) and then add the third dimension that contains the velocity information, therefore making it a complete Range-Azimuth-Doppler 3D radar tensor and forwarding it as the input to the LSTM. This is in contrast with the earlier approaches in the literature, where they first process the tensor using the CFAR algorithm to acquire 2D point clouds that distinguish the real targets from the surrounding clutter. However, this procedure may remove some important information from the original radar signal.

The authors of [125] presented a uniquely designed CNN, which they named RT-Cnet. This network takes as the input both the target-level (i.e., range, azimuth, RCS, and Doppler velocity) and low-level (Range-Azimuth-Doppler data cube) radar data for the multi-class road user’s detection system. The system uses a single radar frame and outputs both the classified radar targets, as well as their object proposal created based on the DBSACAN clustering algorithm [68]. In a nutshell, RT-Cnet performs object classification based on low-level data and the target-level radar data. The inclusion of the low-level data (i.e., speed distribution) improved the road user’s classification against the clustering methods. The object detection task is achieved through a combination of the RT-Cnet and a clustering algorithm that generates the bounding box proposal. A radar target detection scheme based on a four-dimensional space of Range-Doppler-Azimuth and elevation attributes acquired from radar sensors was studied in [126]. Their approach’s main aim was to replace the entire conventional module of detection and beamforming from the classical radar signal processing system.

Furthermore, radar Range-Velocity-Azimuth spectrums generated after 3D-FFT have been applied successfully in many other tasks, like human fall detection [127], human-robot classification [128], and pose estimation [129,130].

### 4.3. Radar Micro-Doppler Signatures

The dynamic moving objects within a radar field of view (FOV) generate a Doppler frequency shift in the returned radar signal, referred to as the Doppler effect. The Doppler effect is proportional to the target velocity. Moving objects consist of moving parts or components that vibrate, rotate, or even oscillate around them, with a different motion to the bulk target motion trajectory. The rotation or vibration of these components may induce an additional frequency on the radar returned signals and create a sideband Doppler velocity known as the micro-Doppler signature. This signature provides an image-like representation that can be utilized potentially for target classification or identification using either machine learning or deep learning algorithms.

Therefore, this motion-induced Doppler modulation may be captured to determine the dynamic nature of objects. Typical examples of micro-Doppler signatures for human walking are the frequency modulation motion induced by swinging components such as the arms and the legs and, also, the motion generated from the rotating propellers of helicopters or unmanned aerial vehicles (UAVs), etc.

The authors of [131] introduced the idea of a micro-Doppler signature and moved on to provide a detailed analysis and the mathematical formulation of different micro-Doppler modulation schemes [132,133]. Ideally, there are various methods for micro-Doppler signature extractions in the literature [134]. The most well-known technique among them is the time–frequency analysis called short-time Fourier transform (STFT). The STFT of a given signal $x\left(t\right)$ is estimated mathematically, as expressed in [132] by:

(16) $$STFT(x(t))=X(t,f)=\int_{-\infty }^{\infty }x(t)w(t-\tau )l^{\ell jwt}dt$$

where $w\left(t\right)$ is the weighting function, and $x\left(t\right)$ is the returned radar signal.

Compared to the standard Range-Velocity FFT, STFTs are calculated by dividing a long-time radar signal into shorter frames of equal lengths and After that, computing the FFT on the segmented frames. This procedure can be exploited to estimate the object’s velocity, representing the various Doppler signatures of the object’s moving parts. There are many methods for extracting radar micro-Doppler signatures with better resolutions than the STFT method; however, discussing them is not within the scope of this work.

Over the last decade, radar-based target classification using micro-Doppler features has gained significant research interest, especially with the recent prevalence of high-resolution radars (like the 77-GHz radar), resulting in much more distinct feature representations. In [135], different deep learning methods were applied to the micro-Doppler signatures obtained from Doppler radar for car, pedestrian, and cyclist classifications. The authors of [136,137] extracted and analyzed the micro-Doppler signature for pedestrian and vehicle classifications. In [138], the micro-Doppler spectrograms of different human gait motions were extracted for human activity classifications. Similarly, the authors of [139] performed both human detection and activity classification, exploiting radar micro-Doppler spectrograms generated from Doppler radar using DCNNs. However, without the range and angle dimensions, their system cannot spatially detect humans but only predict a human presence or absence from the radar signal.

Angelov et al. [38] demonstrated the capability of different DCNNs to recognize cars, people, and bicycles using micro-Doppler signatures extracted from an automotive radar sensor. The authors of [140] presented an approach based on hierarchical micro-Doppler information to classify vehicles into two groups (i.e., wheeled and tracked vehicles). Moreover, P. Molchanov et al. [141] presented an approach to recognized small UAVs and birds using their signatures measured with 9.5-Ghz radar. The features extracted from the signatures were evaluated with SVM classifiers.

### 4.4. Radar Point Clouds

The idea of radar point clouds is derived from computer vision domains concerning 3D point clouds obtained from Lidar sensors. Point clouds are unordered/scattered 3D data representations of information acquired by 3D scanners or Lidar sensors that can preserve the geometric information present in a 3D space and do not require any discretization [142]. This kind of 3D data representation provides high-resolution data that is very rich spatially and contains the depth information, compared to 2D grid image representations. Hence, they are the most commonly used representations for different scene understanding tasks, such as object segmentation, classification, detection, and many more.

Even though radar provides 2D data in polar coordinates, the radar signal can also be represented in the form of point clouds but differently. In conventional radar signal processing, a multi-dimensional 2D-FFT is usually conducted on the reflected radar signals to resolve the range and the velocity of the targets in front of the radar sensor. Later, a CFAR detection is applied to separate the targets from the surrounding clutter and noise. With this approach, the detected peak of the targets after CFAR can be viewed (or represented) as a point cloud with its associated attributes, such as the range, azimuth, RCS, and compensated Doppler velocity. Therefore, a radar point cloud ρ can be defined as a sequence of n=ℵ independent points $p_{i}\in {ℜ}^{d}$, i=1,…,n, in which the order of each point in the point cloud is insignificant. For each radar detection, the radial distance, azimuth angle $\left(\phi \right)$, radar cross-section (RCS), and the ego-motion compensated Doppler velocity can be generated. Therefore a d=4 dimensional radar point cloud is acquired.

Some studies have recently started implementing deep learning models using radar point clouds for different applications [45,49,50,51,143,144]. The authors of [51] presented the first article that employed radar point clouds for semantic segmentation. They used radar point clouds as the input to the classification algorithm, instead of feature vectors acquired from the clustered radar reflections. In essence, they assigned a class label to each of the measured radar reflections. In reference [45], Andreas Danzer et al. employed the PointNet ++ [145] model using radar point clouds for 2D object classifications and bounding box estimations. They used the popularly known PointNets family model, which was ideally designed to consume 3D Lidar point clouds, and adjusted it to fit radar point clouds with different attributes and characteristics.

O. Schumann et al. [50] proposed a new pipeline to segment both static and moving objects using automotive radar sensors for semantic (instance) segmentation applications. They used two separate modules to accomplish their task. In the first module, they employed 2D CNN to segment the static objects using radar grid maps. To achieve that, they introduced a new grid map representation by integrating the radar cross-section (RCS) histogram into the occupancy grid algorithm proposed in [146] as a third additional dimension, which they named the RHG-Layer. In the second module, they introduced another novel recurrent network architecture that accepted radar point clouds as inputs for instance-segmentation of the moving objects. The final results from the two modules were merged at the final stage of the pipeline to create one complete semantic point cloud from the radar reflections. Zhaofei Feng et al. [49] presented object segmentation using radar point clouds and the PointNet ++. Their method explicitly detected and classified the lane marking, guardrail, and moving cars on a highway.

S. Lee [144] presented a radar-only 3D object detection system trained on a public radar dataset based on deep learning. Their work aimed to overcome the lack of enough radar-labeled data that usually led to overfitting in deep learning training. They introduced a novel augmentation method by transforming the Lidar point clouds into radar-like point clouds and adopted Complex-YOLO [147] for one-stage 3D object detection.

### 4.5. Radar Signal Projection

In this method, radar detection or point clouds are usually transformed into a 2D image plane. The relationship between the radar coordinate, the camera coordinate, and the coordinate where the object is situated plays a vital role in this case. Therefore, the camera calibration matrices (i.e., intrinsic and extrinsic parameters) are used to transform the radar points (i.e., detections) from the world coordinate into a camera plane.

In this way, the generated radar image contains the radar detections and its characteristics superimposed on the 2D image grid and, as such, can be applied to deep learning classifiers. Many studies have used these radar signal representations as the input for various deep learning algorithms [28,30,54,58].

Table 1 summarizes the reviewed deep learning-based models employing various radar signal representations for ADAS and autonomous driving applications over the past few years.

### 4.6. Summary

A comprehensive review about radar data processing based on deep learning models is provided, covering different applications such as object classification, detection, and recognition. The study was itemized based on different radar signal representations used as the input to various deep learning algorithms. This is chosen mainly because radar signals are unique, with their own characteristics that are different from other data sources, such as 2D images and Lidar point clouds, which are frequently exploited in deep learning research.

## 5. Deep Learning-Based Multi-Sensor Fusion of Radar and Camera Data

To our best knowledge, no review paper has explicitly focused on the deep learning-based fusion of radar signals and camera information for different challenging tasks involving autonomous driving applications. This makes it somewhat challenging for beginners to venture into this research domain. In this respect, we provide a summary and discussions of the recently published papers according to the new fusion algorithms, fusion architectures, and fusion operations, as well as the datasets published between (2015-current) for the deep multi-sensor fusion of vision and radar information. We also discuss the challenges and possible research directions and potential open questions.

The improved performance achieved by neural networks in processing image-based data has now made some researchers tempted to incorporate additional sensing modalities in the form of multimodal sensor fusion to improve their performance further.

Therefore, by combining more than one sensor, the research community wants to achieve a more accurate, robust, real-time, and reliable performance in any task involved in environmental perceptions for autonomous driving systems. To this end, deep learning models are now being extended to perform deep multi-sensor fusion in order to benefit from the complementarity data from multiple sensing models, particularly in complex environmental situations like an autonomous driving case.

However, most of the recently published articles about DCNN fusion-based algorithms focused on combining camera and Lidar sensor data [31,33,34,35,148].

For instance, reference [31] performed a Multiview 3D (MV3D) object detection by a fusion of the feature representations extracted from three different frames of Lidar and camera—namely, the Lidar bird’s eye view, Lidar front view, and the camera front view. Other studies directly fused point cloud features and image features. Among these studies is Point-fusion [148], which utilizes ResNet [89] and PointNet [149] to generate image features and Lidar point cloud features, respectively, and then uses a global/dense fusion network to fuse them.

Some studies recently considered a neural networks-based fusion of radar signals with camera information to achieve different tasks in autonomous driving applications. The distinctiveness of radar signals and the lack of accessible datasets have contributed to insufficient studies, in that respect. Additionally, this could also be due to the high-sparsity characteristic of radar point clouds acquired with most automotive radars (typically, ≤64 points).

Generally, the challenging task concerning using radar signals with deep learning models is how to model the radar information to suit the required 2D image representation needed by the majority of deep learning algorithms. Many authors have proposed different radar signal representations in this respect, including Range-Doppler-Azimuth maps, radar-grid maps, micro-Doppler signature, radar signal projections, raw radar point clouds, etc. To this end, we itemized our review according to these four fundamental aspects—namely, radar signal representations, fusion levels, deep learning-based fusion architectures, and the fusion operations.

### 5.1. Radar Signal Representations

Radar signals are unique in their peculiar way, as they represent the reflection points obtained from target objects within the proximity of the radar sensor field of view. These reflections are accompanied by their respective characteristics, such as the radial distance, radial velocity, RCS, angle, and the amplitude. Ideally, these signals are 1D signals that cannot be applied directly to DCNN models, which usually require grid map representations as the input for image recognition. Therefore, radar signals are required to be transformed into 2D image-like tensors so that they can be practically deployed together with camera images into deep learning-based fusion networks.

#### 5.1.1. Radar Signal Projection

The radar signal projections technique is when the radar signals (usually, radar point clouds or detections) are transformed into either a 2D image coordinate or into a 3D bird-eye view. Usually, the camera calibration matrices (both intrinsic and extrinsic) are employed to perform the transformation. In this way, a new pseudo-image is obtained that can be consumed by the DCNN algorithms efficiently. A more in-depth discussion about millimeter-wave radar and camera sensor coordinate transformations can be found in [150]. To this end, many deep learning-based fusion algorithms using vision and radar data that are projected onto various domains are reported in the literature [47,48,55,56,57,100,151,152,153,154,155,156].

To alleviate the complex burden involved by the two-stage object detectors with regards to region proposal generations, R. Nabati and H. Qi [47] proposed a radar-based region proposal algorithm for object detection for autonomous vehicles. They generate object proposals and anchor boxes through the mapping of radar detections onto an image plane. By relying on radar detections to obtain region proposals, they avoid the computational steps from the vision-based region proposal method while achieving improved detection results. In order to accurately detect distant objects, reference [54] fused radar and vision sensors. First, the radar image representation was acquired via projecting the radar targets into the image plane and also generating two additional image channels based on the range and radial velocity. After that, they used an SSD model [7] to extract the feature representations from both radar and vision sensors. Lastly, they used a concatenation method to fuse the two features.

The authors of [55], projected sparse radar data onto the camera image’s vertical plane and proposed a fusion method based on a new neural network architecture for object detection. Their framework automatically learned the best level for which the sensor’s data could improve the detection performance. They also introduced a new training strategy, referred to as Black-in, that selected the particular sensor to give preference at a time to achieve better results. Similarly, the authors of [100] performed free space segmentation using an unsupervised deep learning model (GANs) incorporating the radar and camera data in 2D bird-eye view representations. M. Meyer and G. Kuschk [56] conducted a 3D object detection using radar point clouds and camera images based on the deep learning fusion method. They demonstrated how DCNNs could be employed for the low-level fusion of radar and camera data. The DCNNs were trained with the camera images and the BEV images generated from the radar point clouds to detect 3D space cars. Their approach outperformed the Lidar camera-based settings even in a small dataset.

An on-road object detection system for forwarding collision warning was reported in [151] based on vision and radar sensor fusion. Their sensor fusion’s primary purpose is to compensate for a single sensor’s failure encountered in the series fusion architecture by proposing a parallel architecture that relies on each sensor’s confidence index. This approach improves the robustness and detection accuracy of the system. The fusion system consists of three stages: the radar-based object detection, the vision-based object recognition, and the fusion stage based on the radial basis function neural network (RBFNN) that runs in parallel. In [152], the authors performed segmentation using radar point clouds and the Gaussian Mixture Model (GMM) for traffic monitoring applications. The GMM is used as a decision algorithm in the radar point clouds feature vector representation for point-wise classification. Xinyu Zhang et al. [153] presented a radar and vision fusion system for real-time object detection and identification. The radar sensor is first applied to detect the effective target position and velocity information, which is later projected into an image coordinate system of the road image collected simultaneously. An RoI is then generated around the effective target and fed to a deep learning algorithm to locate and identify the target vehicles effectively.

Furthermore, in the work of Vijay John and Seiichi Mita [57], the independent features extracted from radar and monocular camera sensors were fused based on the Yolo object detector in order to detect obstacles under challenging weather conditions. Their sensor fusion framework consists of two input branches to extract the radar and camera-related feature vectors and, also, two output branches to detect and classify obstacles into smaller and bigger categories. In the beginning, radar point clouds are projected onto the image coordinate system, generating a sparse radar image. To reduce the computational burden for real-time applications, the same authors of [57] extended their work and proposed a multitask learning pipeline based on radar and camera deep fusion for joint semantic segmentation and obstacle detection. The network, which they named SO-Net, combined vehicle and free-space segmentations within a single network. SO-Net consists of two input branches that extract the radar and camera features independently, and the two output branches represent the object detection and semantic segmentation, respectively [48].

Similarly, reference [154] adopted the Yolo detector and presented an object detection method based on the deep fusion of mm-wave radar and camera information. They first used the radar information to create a single-channel pseudo-image and then integrate it with an RGB image acquired by the camera sensor to form a four-channel image given to the Yolo object detector as the input.

A novel feature fusion algorithm called SAF (spatial attention fusion) was demonstrated in [58] for obstacle detection based on the mm-wave radar and vision sensor. The authors leveraged the radar point cloud’s sparse nature and generated an attention matrix that efficiently enables data flow into the vision sensor. The SAF feature fusion block contained three convolutional layers in parallel to extract the spatial attention matrix effectively. They first proposed a novel way to create an image-like feature using radar point clouds. They built their object detection fusion pipeline with camera images adapting the fully convolutional one-stage object detection framework (FCOS) [155]. The authors claimed a better average precision performance than the concatenation and element-wise addition fusion approaches, which they suggested were trivial and not suitable for heterogeneous sensors.

Mario Bijelic et al. [156] developed a multimodal adverse weather dataset incorporating a camera, Lidar, radar, gated near-infrared (NIR), and far-infrared (FIR) sensory data to detect adverse weather objects for autonomous driving applications. Moreover, they proposed a novel real-time multimodal deep fusion pipeline that exploited the measurement entropy that adaptively fused the multiple sensory data, thus avoiding the proposal-based level fusion methods. The fusion network adaptively learns to generalize across different scenarios. Similarly, the radar streams and all the other sensory data were all projected into the camera coordinate system.

One of the problems with radar signal transformations exploited by all of the methods mentioned above is that some essential radar information could be lost while conducting the projections. Besides, some of the spatial information from the original radar signals could not be utilized.

#### 5.1.2. Radar Point Clouds

As already discussed in Section 4, millimeter-wave radar signals can be represented as 2D/3D point clouds, just like Lidar sensors, but with different characteristics. They can be applied directly into the Point-Net model [149] for object detection and segmentation. Conventionally, the Point-Net model was designed to consume 3D point clouds obtained with the Lidar sensor for 3D object detection and segmentation. However, the authors of [45] extended the idea with automotive radar data.

To our knowledge, there is no available study in the literature that processed raw radar point clouds with various Point-Net models and proceeded to fuse them with corresponding camera image streams similar to the PointFusion reported in [148]. More studies are needed in this respect.

The main drawback of applying radar point clouds directly to the Point-Net model is losing some basic information by the main radar signal processing chain, as the radar point clouds are obtained through CFAR processing of the raw radar data.

#### 5.1.3. Range-Doppler-Azimuth Tensor

Another way to represent the radar signal is by generating the Range-Doppler-Azimuth tensor using low-level automotive radar information (i.e., the in-phase and quadrature signals (I-Q) of the radar data frames). In this way, a 2D tensor or 3D radar cube can be acquired that can be applied to DCNN classifiers. Accordingly, this is achieved through conducting 2D-FFT on each of the radar data frames to create the Range-Doppler image, with the first FFT performed across each of the columns (range-FFT), generating the range–time matrix. The second FFT is conducted on each of the range bins (Doppler FFT) over the range–time tensor to create the Range-Doppler map, while the third FFT (Angle FFT) is performed on the received antenna dimensions to resolve the direction of arrival.

Recently, some authors performed radar and vision deep neural networks-based fusion by processing low-level radar information to generate Range-Doppler-Azimuth maps [53,153,154]. For instance, reference [53] proposed a new vehicle detection architecture based on the early fusion of radar and camera sensors for ADAS. They processed the radar signals and camera images individually and fused their spatial feature maps at different scales. A spatial transformation branch is applied during the early stage of the network to align the feature maps from each sensor. Regarding the radar signals, the feature maps are generated via processing the 2D Range-Azimuth images acquired from the 3-DFFT on the low-level radar signals instead of the radar point clouds approach that may result in the loss of some contextual information. The feature maps are feed to SSD [7] heads for object detection. The authors showed that their approach can efficiently combine radar signals and camera images and produce a better performance than individual sensors alone.

In reference [98], an object detection pipeline based on radar and vision cross-supervision was presented for autonomous driving under adverse weather conditions. The vision data is first exploited to detect and localize 3D objects. Then, the vision outputs are projected onto Range-Azimuth maps acquired with radar signals for domain supervision of the network. In this way, their model is able to detect objects even with radar signals alone, especially under complex weather conditions in autonomous driving applications, where the vision sensor is bound to fail. A multimodal fusion of radar and video data for human activity classification was demonstrated in [157]. The authors investigated how possible it is to achieve a better performance when the two feature representations from multiple modalities are fused in the early, feature, or decision stage based on a neural network classifier. A single-shot detector is applied to the video data to get the feature vector representations. Simultaneously, micro-Doppler signatures are extracted from the raw radar data in a custom CNN model. However, if radar Range-Doppler-Azimuth images are used with CNNs for classification, the neural networks cannot localize objects or differentiate multiple objects in one image.

### 5.2. Level of Data Fusion

In this section, we review and discuss various studies that employ deep learning networks as means of fusion using radar and vision data according to the level where the two pieces of information are fused.

Conventionally, three primary fusion schemes were designed according to the level where the multi-sensor data merged, including the data level, feature level, and decision level. Subsequently, these schemes are exploited by deep learning-based fusion algorithms in various applications. However, as reported by the authors of [158], neither of the schemes mentioned above can be considered superior in terms of performance.

#### 5.2.1. Data Level Fusion

In data-level fusion (also called low-level), the raw data from radar and vision sensors are fused with deep learning models [47,153,157,158,159,160,161]. It consists of two steps: first, the target objects are predicted with the radar sensor. Then, the predicted object’s region, representing the possible presence of obstacles, is processed with a deep learning framework. Among all the various fusion methods, the low-level fusion scheme is the most computationally expensive approach, as it works directly on the raw sensor data. The authors of [43] proposed an object detection system based on the data-level fusion of radar and vision information. In the first instance, radar detections are relied upon to generate a possible objects region proposal, which is less computationally expensive than the region proposal network generation in two-stage object detection algorithms. Afterward, the ROIs are processed with a Fast R-CNN [105] object detector to obtain the object’s bounding boxes and the classification scores. The authors of [159] proposed object detection and identification based on radar and vision data-level fusion for autonomous ground vehicle navigation. The fusion system was built based on YOLOV3 architecture [10] after mapping the radar detections onto image coordinates. In particular, the radar and vision information were used to validate the presence of a potential target.

X. Zhang et al. [153] proposed an obstacle detection and identification system based on mm-wave radar and vision data-level fusion. MM-wave radar was first used to identify the presence of obstacles and then subsequently create ROIs around them. The ROIs are processed with a R-CNN [104] to realize the real-time obstacle detection and bounding box regression. Similarly, reference [157] employed data-level, feature-level, and decision-level fusion schemes to integrate radar and video data in DCNN networks for human activity classification. Vehicle localization based on the vehicle part-based fusion of camera and radar sensors was also presented in [161]. Deep learning was adopted to detect the target vehicle’s left and right corners.

#### 5.2.2. Feature Level Fusion

In feature level fusion, the extracted features from both radar and vison are combined at the desired stages in deep learning-based fusion networks [53,54,55,56,57,58,162]. In the work of Simon et al. [54], a CNN detection algorithm built based on an SSD detector [7] was applied for the feature-level fusion of radar and vision data. John et al. [57] proposed a deep learning feature fusion scheme by adapting the Yolo object detector [8]. They also demonstrated how their feature-level fusion of radar and vision outperformed the other fusion methods. However, their feature fusion scheme appeared trivial, as the radar point cloud’s sparse characteristics were not considered while creating the sparse radar image. Besides, the two output branches also created additional weights for the network training, which may have eventually led to overfitting.

In [55], a deep learning-based fusion architecture based on camera and radar data was presented for vehicle detection. They fused both radar and camera features extracted with a VGG [88] at a deeper stage of the network. One of the critical innovations about their framework was that they designed it in such a way that it could learn by itself the best depth level where the fusion of the features would result in a better accuracy. A low-level feature fusion was demonstrated based on deep CNNs utilizing radar point clouds and camera images for 3D object detection [56]. They discussed how they obtained a better average precision even with a small dataset compared to the Lidar and vision fusion approach under the same environmental settings. However, they did not use the radar Doppler features as the input of the fusion network. Moreover, in [53], the independent features extracted across radar and camera sensor branches were fused at various scales of the designed network. Then, they applied the SSD heads to the fused feature maps to detect vehicles for ADAS application. They also incorporated a spatial transformation block at the early layers of the network to effectively align the feature maps generated from each sensing branch spatially.

#### 5.2.3. Decision-Level Fusion

For decision-level fusion, the independent detections acquired from the radar and vision modules are fused in the later stage of the deep neural network [157,163]. In [163], a target tracking scheme was proposed using mm-wave radar and a camera DNN-LSTM-based fusion technique. Their proposed fusion technique’s key task was to provide reliable tracking results in situations where either one of the sensors failed. They first located target objects on the image frame and then generated a bounding box around them according to the camera data. Then, they used a deep neural network to acquire the object’s positions according to the bounding box dimensions. The fusion module validated the object positions with those obtained by the radar sensor. Finally, an LSTM was applied to create a continuous target trajectory based on the fused positions. To assist visually impaired people efficiently navigating in a complex environment, Long et al. [164] presented a new fusion scheme using mm-wave radar and an RGB depth sensor, where they employed a mean shift algorithm to process RGB-D images for detecting objects. Their algorithm fused the output obtained from the individual sensors in a particle filter.

### 5.3. Fusion Operations

This section reviewed the deep learning-based fusion frameworks of radar and vision data according to the fusion operation employed.

**Figure 10:**
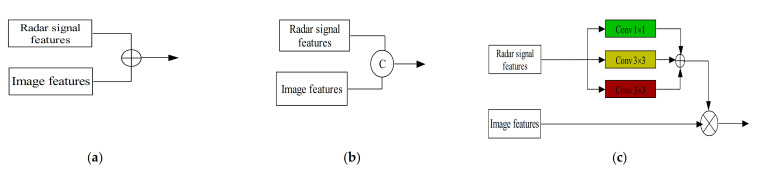
Fusion operation. (**a**) Element-wise addition. (**b**) Concatenation. (**c**) Spatial attention method [58].

According to the different fusion schemes discussed, there should be a particular technique to combine the radar and vision data, specifically for feature-level fusion schemes. The most commonly employed fusion operations are element-wise addition, concatenations, and the spatial attention fusion proposed recently by [58], shown in Figure 10c.

If we denote $M_{i}$ and $N_{j}$ to represents the radar and vison sensor models, while $f^{M_{i}}$ and $f^{N_{j}}$ represent their feature maps at the $l^{th}$ layer of the convolutional neural network, then, for the element-wise addition, the extracted features from the radar and vision are combined to calculate their average mean, as illustrated in [158] and depicted below:

(17) $$f_{l}=H_{l-1}\left(f_{l-1}^{M_{i}}+f_{l-1}^{N_{j}}\right)$$

where $H_{l-1}$ denotes the mathematical function describing the feature transform performed at the $l^{th}$ layer of the network.

For the concatenation operation, the radar and vision feature maps are stacked along the depth dimension. However, at the end of the fully connected layers, the extracted feature maps are typically flattened into vectors before concatenating along their row dimensions. This is illustrated by Equation (18):

(18) $$f_{l}=H_{l-1}\left(f_{l-1}^{M_{i}}\wedge f_{l-1}^{N_{j}}\right)$$

According to most of the papers reviewed, feature concatenation and element-wise addition fusion operations are the most widely employed techniques for radar and vision deep learning-based fusion networks, particularly at the network’s early and middle stages. For example, the authors in [53,54,55,57] both applied feature concatenation operations into their respective systems, while [157] used an element-wise addition scheme.

However, most of the schemes (both element-wise addition and concatenation operations) mentioned earlier do not consider the radar point cloud’s sparse nature while extracting their feature maps. Besides, both of them could be viewed as trivial techniques while tackling heterogeneous sensor features. To overcome that, Shuo Chang et al. [58] proposed a spatial attention fusion (SAF) algorithm utilizing radar and vision information integrated at the feature level. Specifically, the SAF fusion block is a CNN subnetwork consisting of convolutional layers, each with independent receptive fields. They are fed with the radar pseudo-images acquired using radar point clouds to generate the spatial attention information that will be fused with vision feature maps. In this way, the authors obtained promising results for object detection problems.

Moreover, M. Bijelic [156] proposed an adaptive deep fusion scheme utilizing Lidar, RGB camera, gated camera, and radar sensory data. All the multi-sensor data were projected onto a common camera plane. The fusion scheme depends on sensor entropy to extract the features at each exchange block before analyzing them with SSD heads for object detection.

### 5.4. Fusion Network Architectures

This section reviews radar and camera deep learning-based fusion schemes according to the network architectures designed by the various published studies.

Different architectures have been designed purposely in the deep learning domains to suit a particular application or task targeted. For instance, in object detection and related problems, DCNNs are already grouped into either two-stage or one-stage object detectors. Each has its pros and cons toward achieving a better accuracy. In this regard, the neural network-based fusion of radar and vision models for object detection also follows the same network set-up as mentioned above in most of the published articles.

For example, several studies performed radar and vision deep learning-based fusion when building their architectures on top of one-stage object detector [53,54,57,58,154,159] or two-stage object detector models [47,153,156]. However, some studies extended the network structure to capture the peculiarities and more semantic information in radar signals [55]. In order to accommodate the additional pseudo-image channel generated from the radar signals, the authors of [159] built their network structures based on RetinaNet [165] with a VGG backbone [88]. One interesting point about their network architecture is that the network decides the best level to fuse radar and vision data to obtain a better performance. Besides, feature fusion is performed in the deeper layers of the network for optimal results.

Moreover, the authors of [98] built their fusion framework based on 3D autoencoders that consumed the RAmaps snippets as inputs after performing cross-modal supervision with vision detection on them.

**Figure 11:**
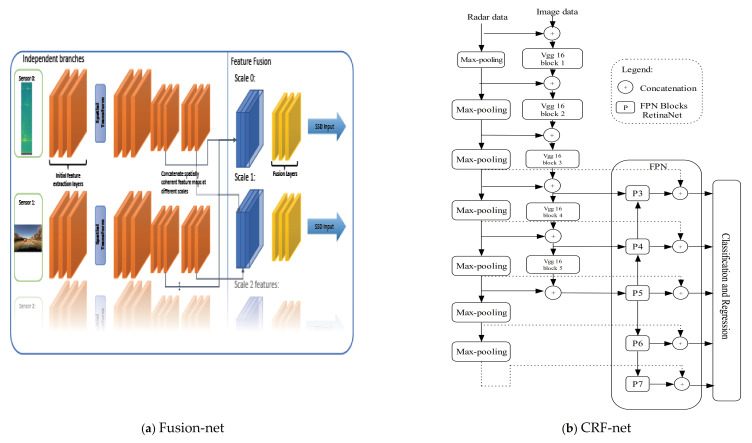

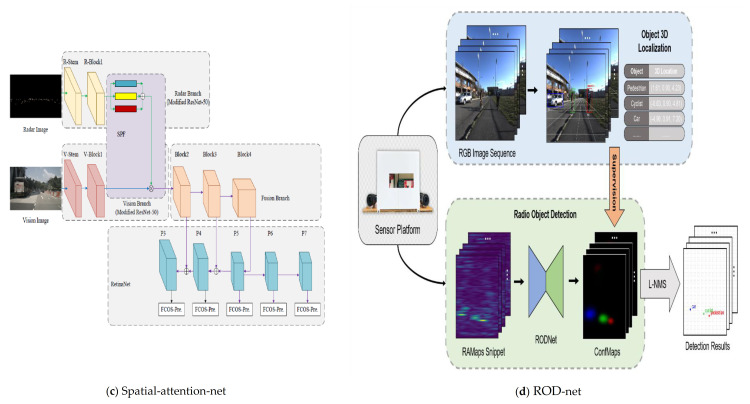
Example of some of the recent deep learning-based fusion architectures exploiting radar and camera data for object detection. (**a**) Fusion-net [53], (**b**) CRF-net [55], (**c**) Spatial fusion-net [58], and (**d**) ROD-net [98].

Figure 11 gives some examples of the recent deep learning-based fusion architectures consuming both radar and vision for object detection and bounding regression. Additionally, we provided a summary of all the reviewed papers in Table 2.

## 6. Datasets

This section provides a review of the radar signal datasets available in the literature, as well as the datasets containing radar data synchronized with other sensors, such as a camera and Lidar, applied in different deep-multimodal fusion systems for autonomous driving applications. Ideally, a massive amount of annotated datasets is necessary in order to achieve the much-needed performance using deep learning models for any tasks in autonomous driving applications.

A high-quality, large-scale dataset is of great importance to the continuous development and improvements of the object recognition, detection, and classification algorithms, particularly in the deep learning domain. Similarly, to train a deep neural network for complex tasks such as those in autonomous driving applications requires a huge amount of training data. Hence, a large-scale, high-quality, annotated real-world dataset containing diverse driving conditions, object sizes, and various degrees/levels of difficulties for benchmarking is required to ensure the network robustness and accuracy against complex driving conditions. However, it is quite challenging to develop a large-scale real-world dataset.

Apart from the data diversity and the size of the training data, the training data quality has a tremendous impact on the deep learning network’s performance. Therefore, to generate high-quality training data for a model, highly skilled annotators are required to label the information (e.g., images) meticulously. Similarly, consistency in providing the model with the necessary high-quality data is paramount in achieving the required accuracy.

Most of the high-performing systems currently use deep learning networks that are usually trained with large-scale annotated datasets to perform tasks such as object detection, object classification, and segmentation using camera images or Lidar point clouds. In essence, several large-scale datasets have been published over the past years (e.g., [162,166,167,168,169]).

However, the vast majority of these datasets provide synchronized Lidar and camera recordings without radar streams. This is despite the strong and robust capabilities of automotive radar sensors, especially in situations where other sensors (e.g., camera and Lidar) are ineffective, such as rainy, foggy, snowy, and strong/weak lighting conditions. Similarly, radars can also estimate the Doppler velocity (relative radial velocity) of obstacles seamlessly. Yet, they are underemployed. Some of the reasons why radar signals are rarely processed with deep learning algorithms could be the lack of publicly accessible datasets, their unique characteristics (which make them difficult to interpret and process), and the lack of ground truth annotations.

In this regard, most earlier studies by many researchers usually self-built their datasets to test their proposed systems. However, creating a new dataset with a radar sensor is time-conscious, and as such, it may take some time to develop a large-scale dataset. Moreover, these datasets and benchmarks are not usually released for public usage. Hence, they do not encourage more research or algorithm developments and comparisons, leaving radar signal-based processing with neural networks miles behind in comparison to camera and Lidar data processing in the computer vision and deep learning domains.

From the beginning of 2019-current, more datasets with radar information are being published [41,42,43,44,52,98,170,171], therefore enabling more research to be realized using high-resolution radars and enhancing the development of multimodal fusion networks for autonomous driving using deep neural network architectures. For example, the authors of [170] developed the Oxford radar dataset for autonomous vehicle and mobile robot applications, benefitting from the FMCW radar sensor’s capabilities in adverse weather conditions. The large-scale dataset is an upgrade to their earlier release [42], incorporating one mm-wave radar and two additional Velodyne 3D Lidars and recorded for over 280 km of urban driving at Central Oxford under different weather, traffic, and lighting conditions. The radar data provided contained range–angle representations without ground truth annotations for scene understanding. Even though they could not release the raw radar observation, the Range-Azimuth data gave some clue into the real radar recordings compared to the 2D point clouds given in reference [41]. However, one of the drawbacks of this dataset was the absence of the ground truth-bounding box annotations.

To facilitate research with high-resolution radar data, M. Meyer and G. Kuschk [43] developed a small automotive dataset with radar, Lidar, and camera sensors for 3D object detection. The dataset provided around 500 synchronized frames with about 3000 correctly labeled 3D objects of ground truth annotations. They provided the 3D bounding box annotations of each sensing model based on Lidar sensor calibrations. However, the size of this dataset was minimal (a few hundred frames), especially for a deep learning model, where a massive amount of data is a prerequisite to performing and avoiding overfitting issues. Similarly, the dataset has limitations concerning different environmental conditions and scenes captured.

Recently, the authors of [44] presented a CARRADA dataset comprising a synchronized camera and low-level radar recordings (Range-Angle and Range-Doppler radar representations) to motivate deep learning communities to utilize radar signals for multi-sensor fusion algorithm developments in autonomous driving applications. The dataset provided three different annotations formats, including sparse points, bounding boxes, and dense masks, to facilitate or explore various supervised learning problems.

The authors of [171], presented a new automotive radar dataset named SCORP that can be applied to deep learning models for open space segmentation. It is the first publicly available radar dataset that includes ADC data (i.e., raw radar I-Q samples) to encourage more research with radar information in deep learning systems. The dataset is available with three different radar representations—namely, Sample-Chirp-Angle tensor (SCA), Range-Azimuth-Doppler tensor (RDA), and DoA tensor (a point cloud representation obtained after polarizing to the Cartesian transformation of the Range-Azimuth (RA) map). Similarly, they used three deep learning-based segmentation architectures to evaluate their proposed dataset using the radar representations mentioned earlier in order to find their effects on the model architecture.

Similarly, the authors of [52] developed a large-scale radar dataset for various objects under multiple situations, including parking lots, campus roads, and freeways. Most importantly, the dataset was collected to complement some challenging scenarios where cameras are usually influenced, such as poor weather and varying lighting conditions. The dataset was evaluated for object classification using their proposed CFAR detector and micro-Doppler classifier (CDMC) algorithm, consisting of two stages: detection and classification. They performed raw radar data processing to generate the object location and Range-Angle radar data cube in the detection part, while, in the classification part, STFFT processing was conducted on the radar data cube, concatenated afterward, and then, it was put forward as the input to the deep learning classifier. They compared the performance of their framework with a small decision tree algorithm using handcrafted features. Even though the dataset was not publicly available at the time of writing this paper, the authors promised to release some portion of it for public usage.

Reference [41] was the first publicly published multimodal dataset captured based on a full $360^{{}^{\circ}}$ sensor suite coverage of radar, Lidar, and cameras with an autonomous vehicle explicitly developed for public road usage. It was also the first dataset that provided radar information, 3D object annotations, and data from nighttime and rainy conditions, as well as object attribute annotations. It had the highest 3D object annotation compared to most of the previously published datasets, like the KITTI dataset [167]. However, this dataset provided only preprocessed nonannotated sparse 2D radar point clouds with few points per frame, ignoring the object’s velocity and textural information in the low-level radar streams. To address that, Y. Wang et al. [98] developed another dataset called CRUW that contained a synchronized stereo vision and radar data frames (Range-Azimuth maps) for different autonomous driving conditions. In particular, the dataset was collected to evaluate their proposed framework for object detection via vision–radar cross-modal supervision under adverse weather. They acknowledged that, by relying purely on radar Range-Azimuth maps, multi-object detection could be enhanced compared to using radar point clouds that ignore the object speed and textural information. The CRUW dataset contains about 400,000 frames of radar and vision information under several autonomous vehicle driving conditions recorded on campuses, in cities, on streets, on highways, and in parking lots.

In order to perform multimodal fusion in complex weather conditions for autonomous vehicles, the authors of [156] developed the first large-scale adverse weather dataset captured with a camera, Lidar, radar, gated NIR, and FIR sensors containing up to 100,000 labels (both 2D and 3D). The dataset contains uncommon complex weather conditions, including heavy fog, heavy snow, and severe rain, collected for about 10,000 km of driving. Similarly, they assessed their novel dataset’s performance by proposing a deep multimodal fusion system that fuses the multimodal data according to the measurement entropy. This contrasts with the proposal-level fusion approach, achieving 8% mAp on hard scenarios, irrespective of the weather conditions. Table 3 summarizes some of the publicly available datasets containing radar data and other sensing modalities.

## 7. Conclusions, Discussion, and Future Research Prospects

Object detection and classification using Lidar and camera data is an established research domain in the computer vision community, particularly with the deep learning progress over the years. Recently, radar signals are being exploited to achieve the tasks above with deep learning models for ADAS and autonomous vehicle applications. They are also applied with the corresponding images collected with camera sensors for deep learning-based multi-sensor fusion. This is primarily due to their strong advantages in adverse weather conditions and their ability to simultaneously measure the range, velocity, and angle of moving objects seamlessly, which cannot be achieved or realized easily with cameras. This review provided an extensive overview of the recent deep learning networks employing radar signals for object detection and recognition. In addition, we also provided a summary of the recent studies exploiting different radar signal representations and camera images for deep learning-based multimodal fusion.

Concerning the reviewed studies about radar data processing on deep learning models, as summarized in Table 1, there is no doubt that a high recognition performance and accuracy have been achieved by the vast majority of the presented algorithms. Notwithstanding, there are some limitations associated with some of them, especially with regards to the different radar signal representations. We provide some remarks about this aspect, as itemized in the follow-up.

The first issue is the radar signal representation. One of the fundamental steps about using radar signals as inputs to deep learning networks is how they can be modeled (i.e., represented) to fit in as the required input of basic deep learning networks. Our review paper was structured according to different radar signal representations, including radar grid maps, Range-Doppler-Azimuth maps, radar signal projections, radar point clouds, and the radar micro-Doppler signature, as depicted in Table 1.

Intuitively, each and every one of these methods has its associated merits and demerits, as it is applied as an input to deep learning algorithms for different applications. For instance, a radar grid map provides a dense map accumulated over a period of time. The occupancy grid algorithm is one of the conventional techniques used to generate radar grid maps, which is a probability depicting the occupancy state of objects. DCNNs can quickly evaluate these maps to determine the classes of objects detected by the radar (e.g., pedestrians, vehicles, or motorcyclists) in an autonomous driving setting. The main drawbacks of this approach are the removal of dynamic moving objects based on their Doppler information. The dense map generated with sparse radar data from the grid map algorithm will contain many empty pixels in the map, creating an additional computational burden in the DCNN feature extractions. Moreover, some raw radar signals may be lost in the course of grid map formation that may likely contribute to the recognition task.

For the Range-Doppler-Azimuth representation, most of the reviewed papers used this approach, as it generally returns 2D image-like tensors illustrating different object energies and amplitudes embedded in their specific positions. One of the fundamental problems of this procedure is the classification of multiple objects in a single scene or image. Similarly, the task of processing the low-level radar data (i.e., raw) might lead to the loss of some essential data that can be vital to the classification problem. The accumulation of more object information based on clustering and multiple radar frames is one way to mitigate this drawback but will add more computational complexity to the system. Recently, reference [125] presented a new architecture that consumes both low-level radar data (Range-Azimuth-Doppler tensor) and target levels (i.e., range, azimuth, RCS, and Doppler information) for multi-class road user detection. Their system takes only a single radar frame and outputs both the target labels and their bounding boxes.

Micro-Doppler signatures are another radar data representation that is also being deployed in various deep learning models to accomplish tasks like target recognition, detection, activity classification, and many more. The signatures depict the energy about the micromotion of the target’s moving components. However, using this signature as the input to deep learning algorithms can only identify an object’s presence or absence in the radar signal. Objects cannot be spatially detected, since the range and angle information are not exploited.

Radar point clouds provide similar information to the popular 3D point clouds obtained with laser sensors. They have since been applied to the existing deep learning architectures designed mainly for Lidar data by many researchers recently. Even though radar data is much noisier, sparser, and with a lot of false alarms, it has demonstrated an encouraging performance that cannot be overlooked entirely, especially in autonomous vehicle applications. However, the overlaying radar signal processing required to acquire radar point clouds might result in the loss of significant information from the raw radar signal. The authors of [125] decided to incorporate both low-level radar and radar point clouds into their proposed network architecture to investigate this issue. The authors of [124] used the 3D Range-Velocity-Azimuth tensor generated with radar spectra as the input to LSTM for vehicle detection, avoiding the CFAR processing approach of obtaining 2D point clouds.

Radar point clouds are also projected onto the image plane or birds-eye view using the coordinate relationships between radar and camera sensors creating pseudo-radar images. As a result, the generated images are used as the input for the deep learning networks. However, to the best of our knowledge, and at the time of writing this paper, we did not come across a study that uses this type of radar signal representation as the input for any deep learning model, whether for detection or classification. Many of the existing papers with this type of radar signal projections were about a multi-sensor fusion of radar and camera. More research should be encouraged in this direction. The main problem with radar signal projections is the loss of some vital information due to the transformation, and the created image usually contained a lot of empty pixels due to the sparsity of the radar point clouds. Camera and radar sensor calibrations are a very challenging and erroneous task that needs to be considered.

Secondly, the lack of large-scale and annotated public radar datasets is a vital issue that has hindered the progress of radar data processing in deep learning models over the years. The majority of the reviewed articles self-recorded their datasets to test their proposed methods, making it difficult for new researchers to compare and evaluate their algorithms, as the datasets are inaccessible. Developing radar datasets is a difficult and time-consuming task. Over the last two years, new datasets have been developed with various kinds of radar signal representations and under different autonomous driving settings and weather conditions.

In general, no literature is available to our knowledge that compared the different radar signal representation performances in a single neural network model to effectively evaluate which one is better and during which particular condition or application. Similarly, none of the available literature considered the fusion of the different radar signal representations into neural networks. We presumed none of those radar signal representations could be viewed as superior, as there were many things involved, including the type of radar sensor used, model designed, the datasets, etc. These problems need to be explored further in the future. Most of the radar data processing in deep learning models is mainly performed using existing network architectures specifically designed to handle other sensory data, particularly camera images or Lidar data. Moreover, most of the models trained with radar data were not trained from scratch; instead, they were trained on top of existing models that were ideally designed for camera or Lidar data (based on transfer learning). New research should be tailored towards designing network architectures that can specifically handle radar signal features and their peculiarities.

Table 2 summarizes the reviewed studies for the deep learning-based multimodal fusion of radar and camera sensors for object detection and classification. Based on the reviewed papers, we can conclude that significant accuracies and performances were attained. However, many aspects need to be improved, including the fusion operations, deep fusion architectures, and the datasets utilized. In the follow-up, we analyzed the reviewed papers and provided some future research directions.

In terms of fusion operations, the most popular approaches like element-wise addition and feature concatenation were regarded by many studies as elementary operations. Even though the authors of [58] presented their approach tackling that by creating a spatial attention network using combinations of filters as one of the fusion operation components, their approach needs to be investigated further using different model architectures to ascertain its robustness. New fusion operations should be designed to accommodate the uniqueness of radar features.

For the fusion architectures, most radar and vision deep learning-based fusion networks are models meant to process Lidar and vision information. They are mostly structured to attained high-accuracy performances, neglecting other important issues like redundancy or if the system can function as desired if one sensor is defective or provides noisy input, besides what the impact or performance of the system is with low/high-sensor inputs. In that respect, new networks should be designed to handle the fusion of radar and camera data specifically and also consider the particular characteristics of radar signals. Similarly, the networks should function efficiently even if one sensor fails or returns noisy information. Other deep learning models such as GANs and autoencoders, as well as RNNs, should be explored for fusion radar and camera data. Another future research prospect could exploit GANs as semi-supervised learning to generate labels from radar and vision data, thus avoiding laborious and erroneous tasks by humans or machines. The complexity of these network architectures also needs to be duly considered for real-time applications.

About the level of fusion, the authors of [158] already demonstrated that none of the available types of fusion-level schemes can be regarded as superior in terms of performance. However, this needs to be investigated further using many network architectures and large-scale datasets.

Developing a large-scale multimodal dataset containing both low-level (I-Q radar data) and target-level data (point clouds) with annotations (both 2D and 3D) under different autonomous driving settings and environmental conditions is a future research problem that ought to be considered. This will enable the research community to evaluate their proposed algorithms with those available in the literature, as most of the earlier studies self-recorded their own and cannot be accessed.

## Figures and Tables

**Table 1:** Summary of the deep-learning algorithms using different radar signal representations.

Reference	Radar Signal Representation	Network Model	Task	Object Type	Dataset	Remarks/Limitation
A. Angelov et al., [38]	Micro-Doppler signatures	CNN and LSTM	Target classification	Car, people, and bicycle	Self-developed	Their dataset is small. Hence, a larger radar dataset is required to train the neural network.
A. Danzer et al., [45]	Radar pointclouds	PointNet [149] and Frustum PointNets [35]	Car detection	Cars	Self-developed	Their dataset is relatively small, with only one radar object per measurement cycle. Besides, it contains a few object classes.
O. Schumann et al., [50]	Radar pointclouds	CNN, RNN	Segmentation and classification of static objects	Car, building, curbstone, pole, vegetation, and other	Self-developed	Their approach needs to be evaluated using a large-scale radar dataset.
O. Schumann et al., [51]	Radar pointclouds.	PointNet++ [145]	Segmentation	Car, truck, pedestrian, pedestrian group, bike, and static object	Self-developed	They used the whole radar point clouds as input, and obtained probabilities for each radar reflection point, thus avoiding the clustering algorithm. No semantic instance segmentation was performed.
S. Chadwick et al., [54]	Radar image	CNN	Distant vehicle detection	Vehicles	Self-developed	They used a very trivial radar image generation that does not consider the sparsity of radar data.
O. Schumann et al., [117]	Radar target clusters	Random forest classifier and LSTM	Classification	Car, pedestrian, bike, truck, pedestrian group, and garbage	Self-developed	Only classes with many samples returned the highest accuracy.
M. Sheeny et al., [122]	Range profile	CNN	Object detection and recognition	Bike, trolley, mannequin, cone, traffic sign, stuffed dog	Self-developed	Their system captured only indoor objects, and they did not make use of the Doppler information.
K. Patel et al., [123]	Range-Azimuth	CNN, SVM, and KNN	Object classification	Construction barrier, motorbike, baby carriage, bicycle, garbage container, car, and stop sign	Self-developed	Their system works on the ROIs instead of the complete rang-azimuth maps. And also the first to the allowed classification of multiple objects with radar data in real scenes.
B. Major et al., [124]	Range-azimuth-Doppler tensor.	CNN	Object detection	Vehicles	Self-developed	They showed how to leveraged the radar signal velocity dimension to improve the detection results
A Palffy et al., [125]	Range-Azimuth and radar Point clouds	CNN	Road users detection	Pedestrians, cyclists, and cars	Self-developed	They are the first to utilized both low-level and target-level radar data to addressed moving road user detection.
D. Brodeski et al., [126]	Range-Doppler-Azimuth-Elevation	CNN	Target detection	2-Classes object, and non-object	Self-built (in the anechoic chamber)	Real-world data was not included.
Y. Kim and T. Moon [139]	Micro-Doppler signatures	CNN	Human detection and activity classification	Human, dog, horse, and car	Self-developed	Their system could only detect humans presence or absence in the radar signal since there is no range and angle dimensions.
S. Lee [144]	Bird-eye- view	3D object detection	Cars	Cars	Astyx HiRes [39]	Radar Doppler information was not incorporated into the network.

**Table 2:** A summary of deep learning-based fusion methods using radar and vision data.

Reference	Sensors	Sensors Signal Representation	Network Architecture	Level of Fusion	Fusion Operation	Problem	Object Type	Dataset
R. Nabati and H. Qi [47]	Radar and visual camera	RGB image and radar signal projections	Fast-R-CNN (Two-stage)	Mid-level fusion	Region proposal	Object detection	2D vehicle	Nuscenes [41]
V. John et al., [48]	Radar and camera	RGB image and radar signal projections	Yolo object detector (Tiny Yolov3), and, Encoder-decoder	Feature level	Feature concatenation	Vehicle Detection and Free space Segmentation	Vehicles and free space	Nuscenes [41]
L.Teck-Yian et al., [53]	Radar and camera	RGB image and Radar Range-Azimuth image	Modified SSD With two branches each for one sensor	Early level fusion	Feature concatenation	Detection and classification	3D vehicles	Self-recorded
S. Chadwick et al., [54]	Radar and visual camera	RGB image and Radar range-velocity maps	One-stage detector	Middle	Feature concatenation and addition	Object detection	2D vehicle	Self-recorded
F. Nobis et al. (CRF-Net), [55]	Radar and visual camera	RGB image and radar signal projections	RetinaNetwith a VGG backbone	Deeper layers	Feature concatenated	Object detection	2D road vehicles	NuScenes [41]
Meyer and Kuschk [56]	Radar and visual camera	RGB image and radar point clouds	Faster RCNN (Two-stage)	Early and Middle	Average Mean	Object Detection	3D vehicle	Astyx hiRes 2019 [43]
Vijay John and Seiichi Mita [57]	Radar and camera	RGB image and radar signal projections	Yolo object detector (Tiny Yolov3)	Feature level(late)	Feature concatenation	2D image-based obstacle detection	vehicles, pedestrians, two-wheelers, and objects (movable objects and debris)	Nuscenes [41]
S. Chang et al., [58]	Radar and camera	RGB image and radar signal projections	Fully convolutional one-stage object detection framework (FCOS)	Feature level	spatial attention feature fusion (SAF)	Obstacle detection	Bicycle, car, motorcycle, bus, train, truck	Nuscenes [41]
W.Yizhou et al.(RODnet), [98]	Radar and Stereo videos	2D image and Radar Range-Azimuth maps	3D autoencoder, 3D stacked hourglass, and 3D stacked hourglass with temporal inception layers	Mid level	Cross-modal learning and supervision	Object detection	Pedestrians, cyclists, and cars.	CRUW [98]
V. Lekic and Z. Babic [100]	Radar and visual camera	RGB image and Radar grid maps	GANs (CMGGAN model)	Mid-level	Feature fusion and semantic fusion	Segmentation	Free space	Self-recorded
Mario Bijelic et al., [156]	Camera, lidar, radar, and gated NIR sensor	Gated image, RGB image, Lidar projection, and radar projection	Modified VGG [88] backbone, and SSD blocks	Early feature fusion (Adaptive fusion steered by entropy)	Feature concatenation	Object detection	Vehicles	A novel multimodal dataset in adverse weather dataset [156]
Richard J. de Jong [157]	Radar and camera	RGB image and Radar micro-Doppler spectrograms	CNN	Data, middle and feature level fusion	Feature concatenation	Human Activity Classification	Walking person	Self-recorded

**Table 3:** A summary of the available datasets containing radar data and/without other sensors data.

Dataset	Sensing Modalities	Size	Scenes	Labels	Frame Rate	Radar Signal Representation	Type of Objects	Recording Condition	Recording Location	Published Year	Availability/LINK
Nuscenes [41]	Visual Cameras (6), 3D Lidar, and Radars (5)	1.4 frames (Cameras and Radars) and 390 K frames of Lidar	1 K	2D/3D bounding boxes (1.4M)	1 HZ/10 HZ	2D radar point clouds	23 object classes	Nightime/rain and light weather	Boston, Singapore.	2019	Public, (https://www.nuscenes.org/download (accessed on 20 January 2021))
Astyx HiRes [43]	Radar, visual cameras, and 3D Lidar	500 frames	n.a	3D bounding boxes	n.a.	3D Radar point clouds	Car, bus, motorcycle, person, trailer, and truck	Daylight	n.a.	2019	Public, (http://www.astyx.netaccessed on 30 December 2020))
CARRADA [44]	Radar and Visual camera	12,726 total number of frames	n.a	Sparse point, bounding boxes, and dense masks.	n.a.	Range-angle and range-Doppler raw radar data	cars, pedestrians, and cyclist	n.a.	Canada	2020	To be released
[52]	Radar	n.a.	Parking lot, campus road, city road and, freeway	Metadata (Object location and class)	30 fps	Raw radar I-Q samples	Pedestrian, cyclist, and cars	Under challenging light conditions	n.a.	2019	To be released via (https://github.com/yizhou-wang/UWCR (accessed on 5 December 2020)
CRUW [98]	Stereo cameras and 77GHz FMCW Radars (2)	More than 400 K frames	Campus road, city street, highway, parking lot, etc	Annotations (Object location and class)	30 fps	Range-Azimuth maps (RAMaps)	About 260 K objects	Different autonomous driving scenarios, including dark, strong light, and blur	n.a	2020	Self-collected
[156]	Cameras(2), Lidars(2), radar, gated NIR, and FIR	1.4 M frames	10,000 km of driving	2D/3D (each 100k)	10 Hz	Radar signal projections	n.a	Clear, nighttime, dense fog, light fog, rain, and snow	Northern Europe (Germany, Sweden, Denmark, and Finland)	February and December 2019	On request
Oxford Robot-Car [170]	Visual cameras (6), 2D & 3D Lidars (4), GNSS, Radar, and Inertial sensors	240 k (Radar), 2.4 frames (Lidar) and 11,070,651 frames (Stereo camera	n.a.	No	n.a.	Range-Azimuth maps	Vehicles and pedestrians	Direct sunlight, heavy rain, night, and snow	Oxford	2017, 2020	Public, (https://ori.ox.ac.uk/oxford-radar-robotcar-dataset (accessed on 7 January 2021))
SCORP [171]	Radar and Visual camera	3913 frames	11 Driving sequences	Bounding boxes.	n.a.	SCA, RDA, and DoA tensors	n.a.	n.a.	n.a.	2020	Public, (https://rebrand.ly/SCORP (accessed on 27 November 2020))
